# Socioeconomic status and stroke incidence, prevalence, mortality, and worldwide burden: an ecological analysis from the Global Burden of Disease Study 2017

**DOI:** 10.1186/s12916-019-1397-3

**Published:** 2019-10-24

**Authors:** Abolfazl Avan, Hadi Digaleh, Mario Di Napoli, Saverio Stranges, Reza Behrouz, Golnaz Shojaeianbabaei, Amin Amiri, Reza Tabrizi, Naghmeh Mokhber, J. David Spence, Mahmoud Reza Azarpazhooh

**Affiliations:** 1 Department of Neurology, Ghaem Hospital, School of Medicine, Mashhad University of Medical Sciences, Mashhad, Iran; 2 Neurobiology Research Center, Shahid Beheshti University of Medical Sciences, Tehran, Iran; 3 Department of Neurology and Stroke Unit, San Camillo de’ Lellis General District Hospital, Rieti, Italy; 4 Department of Epidemiology and Biostatistics, Schulich School of Medicine & Dentistry, Western University, London, Ontario Canada; 5 Department of Family Medicine, Schulich School of Medicine & Dentistry, Western University, London, Ontario Canada; 6 Department of Population Health, Luxembourg Institute of Health, Strassen, Luxembourg; 7 Stroke Program, Department of Neurology, School of Medicine, University of Texas Health Science Center, San Antonio, TX USA; 8 Health Policy Research Center, Institute of Health, Shiraz University of Medical Sciences, Shiraz, Iran; 9 Clinical Neurology Research Center, Shiraz University of Medical Sciences, Shiraz, Iran; 10 Department of Psychiatry and Behavioural Neurosciences, Western University, London, Ontario Canada; 11 Department of Psychiatry, Mashhad University of Medical Sciences, Mashhad, Iran; 12 Stroke Prevention & Atherosclerosis Research Centre, Robarts Research Institute, Western University, Siebens-Drake Building, 1400 Western Rd, London, Ontario N6G 2V4 Canada; 13 Department of Clinical Neurological Science, Western University, London, Ontario Canada; 14 Division of Clinical Pharmacology, Western University, London, Ontario Canada

**Keywords:** Stroke, Cause of death, Global burden of disease, Global health, Non-communicable diseases, Public health practice, Risk factors, Socioeconomic factors, Life style, Morbidity

## Abstract

**Background:**

Socioeconomic status (SES) is associated with stroke incidence and mortality. Distribution of stroke risk factors is changing worldwide; evidence on these trends is crucial to the allocation of resources for prevention strategies to tackle major modifiable risk factors with the highest impact on stroke burden.

**Methods:**

We extracted data from the Global Burden of Diseases, Injuries, and Risk Factors Study (GBD) 2017. We analysed trends in global and SES-specific age-standardised stroke incidence, prevalence, mortality, and disability-adjusted life years (DALYs) lost from 1990 to 2017. We also estimated the age-standardised attributable risk of stroke mortality associated with common risk factors in low-, low-middle-, upper-middle-, and high-income countries. Further, we explored the effect of age and sex on associations of risk factors with stroke mortality from 1990 to 2017.

**Results:**

Despite a growth in crude number of stroke events from 1990 to 2017, there has been an 11.3% decrease in age-standardised stroke incidence rate worldwide (150.5, 95% uncertainty interval [UI] 140.3–161.8 per 100,000 in 2017). This has been accompanied by an overall 3.1% increase in age-standardised stroke prevalence rate (1300.6, UI 1229.0–1374.7 per 100,000 in 2017) and a 33.4% decrease in age-standardised stroke mortality rate (80.5, UI 78.9–82.6 per 100,000 in 2017) over the same time period. The rising trends in age-standardised stroke prevalence have been observed only in middle-income countries, despite declining trends in age-standardised stroke incidence and mortality in all income categories since 2005. Further, there has been almost a 34% reduction in stroke death rate (67.8, UI 64.1–71.1 per 100,000 in 2017) attributable to modifiable risk factors, more prominently in wealthier countries.

**Conclusions:**

Almost half of stroke-related deaths are attributable to poor management of modifiable risk factors, and thus potentially preventable. We should appreciate societal barriers in lower-SES groups to design tailored preventive strategies. Despite improvements in general health knowledge, access to healthcare, and preventative strategies, SES is still strongly associated with modifiable risk factors and stroke burden; thus, screening of people from low SES at higher stroke risk is crucial.

**Electronic supplementary material:**

The online version of this article (10.1186/s12916-019-1397-3) contains supplementary material, which is available to authorized users.

## Background

Among 240 causes of death, stroke is globally the second cause of death after ischaemic heart disease [1], and it is projected to remain so by 2030 [2]. This rank varies slightly across low-income countries (LICs), lower-middle-income countries (LMICs), upper-middle-income countries (UMICs), and high-income countries (HICs) as classified by the World Bank (Table 1) [3]. Further, stroke survivors may suffer from disabilities, requiring temporary or lifelong assistance, resulting in an enormous burden, both in human and economic costs. Evidence suggests that socioeconomic deprivation is not only associated with stroke and its risk factors, but also increases stroke severity [4] and mortality [5], and stroke incidence at younger ages [4].

**Table 1:**
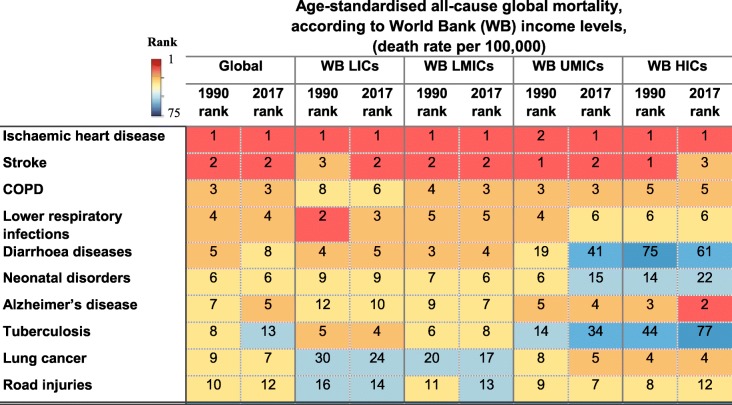
Age-standardised all-cause global mortality rank in 1990 and 2017 being classified by World Bank income levels (extracted from [8])

*HICs* high-income countries, *LICs* low-income countries, *LMICs* lower-middle-income countries, *UMICs* upper-middle-income countries, *WB* The World Bank

**Fig. 1:**
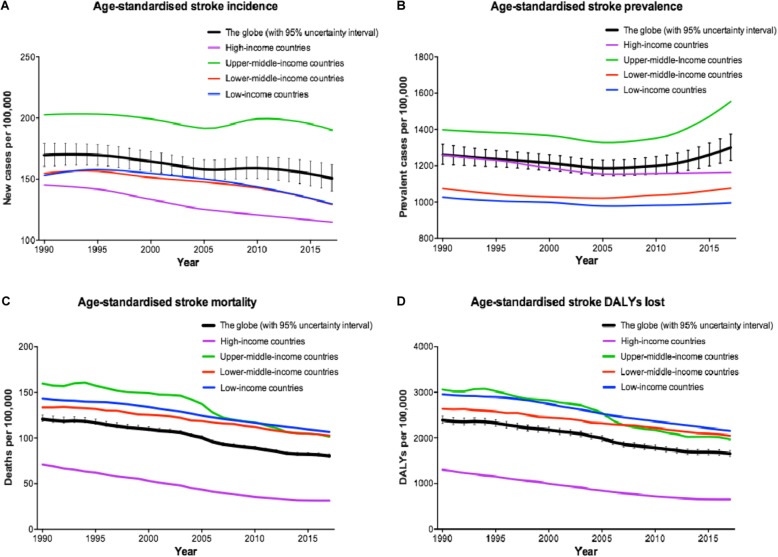
Trends in age-standardised stroke **a** incidence, **b** prevalence, **c** mortality, and **d** disability-adjusted life years (DALYs) lost from 1990 to 2017 in different regions being classified as to the World Bank income levels (extracted from [8])

Distribution of stroke risk factors in the context of socioeconomic status is changing worldwide (SES; Additional file 1: Text S1); understanding these trends is helpful in reducing the risk, through allocation of resources to those modifiable risk factors with the highest impact on stroke (Fig. 1). From 1990 to 2010, the age-standardised incidence of stroke decreased significantly by 12% in HICs, while in LMICs, it increased, although non-significantly, by about 12% [6]. However, mortality rates decreased significantly in both groups of countries (mean 37% in HICs vs. 20% in LMICs), of which 31% (with about 80% of it in LMICs) were in children and young adults (below 65 years) [6]. Further, the available evidence indicates that almost 90% of cardiovascular disease, including stroke and myocardial infarction, is caused by potentially modifiable risk factors [7]. We aimed to study the global and SES-specific stroke incidence, prevalence, burden, and mortality and to estimate the role of age, sex, and modifiable risk factors in stroke mortality.

## Methods

We extracted data from the Global Burden of Diseases, Injuries, and Risk Factors Study (GBD) 2017, coordinated by the Institute for Health Metrics and Evaluation, as of 19 May 2019 [8]. We reported global and SES-specific rates of age-standardised and age-specific stroke incidence, prevalence, mortality, and disability-adjusted life years (DALYs) lost per 100,000 population with 95% uncertainty interval (UI) based on the 25th and 975th values of the ordered 1000 draws between 1990 and 2017. The method for estimating the parameters is explained elsewhere [9–11]. In summary, the GBD enterprise originated from the 1990 World Bank study that was commissioned to measure the status of global health. It comprises information from multiple sources including multiple databases such as MEDLINE, EMBASE, LILACS, Scopus, PubMed and Science Direct, Global Health Database, WHO library and WHO regional databases, VR systems, sample registration systems, household surveys (complete birth histories, summary birth histories, sibling histories), censuses (summary birth histories, household deaths), and Demographic Surveillance Sites (DSS). DisMod-MR 2.0 was used as a meta-regression tool to pool the case fatality data and generate location-year-age-sex-specific case fatality rate estimates.

SES was defined based on the gross national per capita income, as classified by the World Bank (i.e. low-income, lower-middle-income, upper-middle-income, or high-income) [3], and the Socio-Demographic Index (SDI; i.e. low-SDI, low-middle-SDI, middle-SDI, high-middle-SDI, or high-SDI) being developed in the GBD 2016. We also retrieved ranks of ten leading causes of death and stroke-related death attributable to common modifiable risk factors in those regions. We calculated rates and proportions of the abovementioned factors in both and each sexes of different socioeconomic regions from 1990 to 2017. We assessed proportions of the incidence, prevalence, burden, and mortality of stroke in different age groups. Unless explicitly mentioned otherwise, all rates were age-standardised using the GBD standard and reported per 100,000 population [12]. We used Numbers (version 3.6.2 for Mac OS X, Apple Inc., USA) for data compilation, preliminary analyses, and making tables. We used Prism (version 6.0 h for Mac OS X, GraphPad Software Inc.) for analysis and making the graphs.

Thirty-one LICs include Afghanistan, Benin, Burkina Faso, Burundi, Central African Republic, Chad, Comoros, Democratic Republic of the Congo, Eritrea, Ethiopia, Guinea, Guinea-Bissau, Haiti, Liberia, Madagascar, Malawi, Mali, Mozambique, Nepal, Niger, North Korea, Rwanda, Senegal, Sierra Leone, Somalia, South Sudan, Tanzania, The Gambia, Togo, Uganda, and Zimbabwe; 52 LMICs include Angola, Armenia, Bangladesh, Bhutan, Bolivia, Cambodia, Cameroon, Cape Verde, Congo, Côte d’Ivoire, Djibouti, Egypt, El Salvador, Federated States of Micronesia, Georgia, Ghana, Guatemala, Honduras, India, Indonesia, Jordan, Kenya, Kiribati, Kyrgyzstan, Laos, Lesotho, Mauritania, Moldova, Mongolia, Morocco, Myanmar, Nicaragua, Nigeria, Pakistan, Palestine, Papua New Guinea, Philippines, Sao Tome and Principe, Solomon Islands, Sri Lanka, Sudan, Swaziland, Syria, Tajikistan, Timor-Leste, Tunisia, Ukraine, Uzbekistan, Vanuatu, Vietnam, Yemen, and Zambia; 54 UMICs include Albania, Algeria, American Samoa, Argentina, Azerbaijan, Belarus, Belize, Bosnia and Herzegovina, Botswana, Brazil, Bulgaria, China, Colombia, Costa Rica, Croatia, Cuba, Dominica, Dominican Republic, Ecuador, Equatorial Guinea, Fiji, Gabon, Grenada, Guyana, Iran, Iraq, Jamaica, Kazakhstan, Lebanon, Libya, Macedonia, Malaysia, Maldives, Marshall Islands, Mauritius, Mexico, Montenegro, Namibia, Panama, Paraguay, Peru, Romania, Russian Federation, Saint Lucia, Saint Vincent and the, Grenadines, Samoa, Serbia, South Africa, Suriname, Thailand, Tonga, Turkey, Turkmenistan, and Venezuela; and 58 HICs include Andorra, Antigua and Barbuda, Australia, Austria, Bahrain, Barbados, Belgium, Bermuda, Brunei, Canada, Chile, Cyprus, Czech Republic, Denmark, Estonia, Finland, France, Germany, Greece, Greenland, Guam, Hungary, Iceland, Ireland, Israel, Italy, Japan, Kuwait, Latvia, Lithuania, Luxembourg, Malta, Netherlands, New Zealand, Northern Mariana Islands, Norway, Oman, Poland, Portugal, Puerto Rico, Qatar, Saudi Arabia, Seychelles, Singapore, Slovakia, Slovenia, South Korea, Spain, Sweden, Switzerland, Taiwan, The Bahamas, Trinidad and Tobago, United Arab Emirates, the UK, Uruguay, Virgin Islands, and the USA.

## Results

### Stroke incidence, prevalence, mortality, and burden

Based on the GBD 2017 [8], the global crude number of new stroke events has increased by 76% (UI 71–80%) from 6.8 (UI 6.4–7.2) million new events in 1990 to 11.9 (UI 11.1–12.8) million in 2017 (Table 2). However, the age-standardised global stroke incidence rate (i.e. new stroke events per 100,000 population) decreased overall by 11% (UI 9–12%; − 15% in LICs, − 16% in LMICs, − 6% in UMICs, and − 21% in HICs) during the same period of time. The age-standardised global rate of new strokes became 150.5 (UI 140.3–161.8) per 100,000 in 2017. This decrease could be partly explained by more aggressive preventive measures and control of the risk factors. Nevertheless, despite a doubling of the global number of new ischaemic strokes from 1990 to 2017, there was no significant change in its age-standardised rate, while haemorrhagic events have significantly decreased globally and regionally during this period. Ischaemic strokes and intracerebral (not subarachnoid) haemorrhage seem to be more common in males than females; female to male ratio has decreased by 6% from 1990 to 2017.

**Table 2:** Global and regional stroke incidence, prevalence, mortality, and burden from 1990 to 2017

Measure/region	Age	Metric name	All-type stroke			Ischaemic stroke			Intracerebral haemorrhage			Subarachnoid haemorrhage		
			Mean (95% uncertainty interval)			Mean (95% uncertainty interval)			Mean (95% uncertainty interval)			Mean (95% uncertainty interval)		
			1990	2017	Percent change	1990	2017	Percent change	1990	2017	Percent change	1990	2017	Percent change
Incidence														
Global	Age-standardised	Rate	169.6 (160.3 to 179.4)	150.5 (140.3 to 161.8)	− 11 (− 13 to − 9)	98.7 (89.9 to 108.4)	98 (88.1 to 109.7)	− 1 (− 4 to 3)	56.9 (53.6 to 60.4)	39.3 (36.2 to 42.9)	− 31 (− 33 to − 29)	14.1 (12.4 to 16.4)	13.2 (11.8 to 14.6)	− 7 (− 15 to − 4)
		Female to male ratio	0.89	0.84	− 6	0.90	0.83	− 8	0.79	0.73	− 8	1.30	1.31	1
	All ages	Number (million)	6.8 (6.4 to 7.2)	11.9 (11.1 to 12.8)	76 (71 to 80)	3.9 (3.5 to 4.3)	7.7 (7 to 8.7)	101 (94 to 108)	2.3 (2.2 to 2.5)	3.1 (2.9 to 3.4)	34 (30 to 39)	0.6 (0.5 to 0.7)	1.1 (1 to 1.2)	73 (55 to 79)
		Rate	125.9 (118.9 to 133.6)	156.2 (145.5 to 167.9)	24 (21 to 27)	71.4 (64.6 to 78.9)	101.3 (91 to 113.6)	42 (37 to 47)	43.2 (40.6 to 45.9)	40.9 (37.6 to 44.7)	− 5 (− 8 to − 2)	11.4 (10 to 13.4)	13.9 (12.5 to 15.5)	22 (10 to 26)
World Bank low income	Age-standardised	Rate	152.9 (144.6 to 162)	129.7 (120.7 to 139.8)	− 15 (− 17 to − 13)	78.1 (70.6 to 86.5)	75 (66.5 to 85)	− 4 (− 7 to − 1)	61.2 (56.8 to 65.7)	41.4 (37.7 to 45.3)	− 32 (− 35 to − 30)	13.6 (12 to 15.4)	13.4 (11.8 to 15.1)	− 2 (− 7 to − 0)
		Female to male ratio	0.97	0.95	− 2	1.04	0.97	− 7	0.85	0.83	− 1	1.18	1.20	2
	All ages	Number (million)	0.2 (0.2 to 0.3)	0.4 (0.4 to 0.4)	69 (65 to 73)	0.1 (0.1 to 0.1)	0.2 (0.2 to 0.3)	92 (86 to 99)	0.1 (0.1 to 0.1)	0.1 (0.1 to 0.1)	33 (29 to 38)	0 (0 to 0)	0 (0 to 0.1)	98 (83 to 104)
		Rate	71.6 (67.6 to 76.1)	59.7 (55.6 to 64.5)	− 17 (− 19 to − 15)	34.7 (31.3 to 38.8)	33 (29.3 to 37.5)	− 5 (− 8 to − 2)	29.4 (27.3 to 31.5)	19.4 (17.6 to 21.3)	− 34 (− 36 to − 32)	7.5 (6.6 to 8.6)	7.4 (6.5 to 8.3)	− 2 (− 9 to 1)
World Bank lower middle income	Age-standardised	Rate	154.7 (146.1 to 163.7)	129.3 (120.6 to 139.1)	− 16 (− 18 to − 14)	87.5 (79.4 to 96.8)	78.7 (70.3 to 88.8)	− 10 (− 13 to − 7)	54.8 (51.2 to 58.7)	39.2 (35.8 to 42.9)	− 28 (− 31 to − 26)	12.4 (10.9 to 14.5)	11.4 (10.1 to 12.8)	− 8 (− 15 to − 6)
		Female to male ratio	0.99	0.93	− 6	1.04	0.94	− 10	0.86	0.85	− 1	1.23	1.21	− 2
	All ages	Number (million)	1.6 (1.5 to 1.7)	2.9 (2.7 to 3.1)	77 (73 to 82)	0.9 (0.8 to 1)	1.7 (1.5 to 1.9)	96 (89 to 103)	0.6 (0.6 to 0.7)	0.9 (0.8 to 1)	49 (44 to 54)	0.2 (0.1 to 0.2)	0.3 (0.3 to 0.3)	86 (69 to 91)
		Rate	85 (80.2 to 90.4)	93.2 (86.8 to 100.5)	10 (7 to 13)	45.3 (40.7 to 50.4)	54.8 (48.8 to 62.3)	21 (17 to 25)	31.6 (29.5 to 33.8)	29.2 (26.6 to 32)	− 8 (− 11 to − 5)	8.1 (7 to 9.6)	9.3 (8.2 to 10.5)	15 (4 to 18)
World Bank upper middle income	Age-standardised	Rate	202.7 (191.8 to 214.5)	190.1 (176.6 to 205)	− 6 (− 9 to − 3)	114.3 (104.1 to 125.7)	128.7 (116.1 to 143.5)	13 (8 to 17)	75.7 (71.4 to 80.3)	50 (46 to 54.4)	− 34 (− 36 to − 32)	12.7 (10.9 to 16.2)	11.4 (10.2 to 12.7)	− 10 (− 26 to − 6)
		Female to male ratio	0.88	0.78	− 11	0.91	0.80	− 12	0.79	0.67	− 15	1.23	1.25	1
	All ages	Number (million)	3 (2.9 to 3.2)	6.1 (5.6 to 6.6)	101 (94 to 107)	1.7 (1.5 to 1.8)	4.1 (3.7 to 4.6)	148 (138 to 159)	1.2 (1.1 to 1.2)	1.6 (1.5 to 1.7)	38 (33 to 43)	0.2 (0.2 to 0.3)	0.4 (0.3 to 0.4)	74 (42 to 84)
		Rate	143.9 (135.8 to 152.5)	231.5 (214.4 to 249.9)	61 (55 to 66)	78.7 (71.3 to 87.1)	156.6 (141.2 to 175.4)	99 (91 to 107)	55 (51.8 to 58.6)	60.8 (56 to 66.4)	10 (6 to 15)	10.1 (8.7 to 13.1)	14.1 (12.5 to 15.8)	40 (14 to 47)
World Bank high income	Age-standardised	Rate	145.2 (136.8 to 153.9)	114.7 (107.3 to 123)	− 21 (− 23 to − 19)	92.2 (84.1 to 100.9)	74.2 (67.5 to 82.8)	− 19 (− 22 to − 16)	35 (33.1 to 37.2)	21.6 (19.9 to 23.4)	− 38 (− 41 to − 36)	18 (16.1 to 20.1)	18.9 (17.2 to 20.8)	5 (2 to 8)
		Female to male ratio	0.82	0.85	4	0.79	0.79	0	0.73	0.72	− 1	1.36	1.44	6
	All ages	Number (million)	1.8 (1.7 to 2)	2.4 (2.3 to 2.6)	33 (29 to 37)	1.2 (1.1 to 1.3)	1.6 (1.5 to 1.8)	38 (32 to 43)	0.4 (0.4 to 0.5)	0.5 (0.4 to 0.5)	5 (0 to 10)	0.2 (0.2 to 0.2)	0.4 (0.3 to 0.4)	61 (56 to 66)
		Rate	184.6 (173.3 to 195.9)	205.7 (191.8 to 220.3)	11 (9 to 15)	118.3 (107.6 to 129.9)	137 (124.1 to 152.3)	16 (11 to 21)	44.4 (41.9 to 47.3)	39.2 (36.1 to 42.6)	− 12 (− 16 to − 8)	21.8 (19.5 to 24.4)	29.5 (26.9 to 32.4)	35 (31 to 39)
Prevalence														
Global	Age-standardised	Rate	1261 (1208.2 to 1318.7)	1300.6 (1229 to 1374.7)	3 (1 to 5)	942.5 (891.1 to 999.6)	1038 (968.8 to 1114.1)	10 (7 to 13)	260.7 (242.3 to 279.9)	220.2 (199.3 to 241.7)	− 16 (− 19 to − 12)	121.8 (112.6 to 131.9)	113.9 (104.2 to 125.2)	− 6 (− 8 to − 5)
		Female to male ratio	1.02	0.96	−6	0.95	0.90	− 5	1.07	1.00	− 6	1.48	1.46	− 1
	All ages	Number (million)	53.3 (51.1 to 55.7)	104.2 (98.5 to 110.1)	95 (91 to 100)	39 (36.8 to 41.3)	82.4 (77 to 88.5)	112 (106 to 117)	11.9 (11.1 to 12.8)	17.9 (16.2 to 19.7)	50 (43 to 58)	5.4 (5 to 5.9)	9.3 (8.5 to 10.2)	72 (68 to 76)
		Rate	988 (947.7 to 1032.7)	1363.5 (1288.6 to 1441.3)	38 (35 to 41)	722.3 (682.7 to 765.3)	1078.7 (1007.4 to 1158.5)	49 (45 to 53)	221.2 (205.5 to 237.5)	234.5 (211.9 to 257.4)	6 (1 to 11)	100.5 (93.2 to 109.1)	122 (111.6 to 134.1)	21 (19 to 24)
World Bank low income	Age-standardised	Rate	1026.6 (984.2 to 1074.5)	996 (949.6 to 1049.3)	− 3 (− 5 to − 1)	713.4 (672 to 759.5)	726.4 (682.2 to 777.2)	2 (0 to 3)	263.5 (247.6 to 280.1)	220.5 (198.4 to 244.3)	− 16 (− 22 to − 10)	104 (97.1 to 111.6)	102.7 (94.6 to 112.1)	− 1 (− 4 to 2)
		Female to male ratio	1.13	1.08	− 5	1.14	1.06	− 7	1.03	1.04	0	1.32	1.34	2
	All ages	Number (million)	1.7 (1.7 to 1.8)	3.3 (3.2 to 3.5)	91 (87 to 95)	1.2 (1.1 to 1.3)	2.4 (2.2 to 2.5)	100 (97 to 103)	0.5 (0.5 to 0.6)	0.9 (0.8 to 1)	70 (58 to 81)	0.2 (0.2 to 0.2)	0.4 (0.4 to 0.4)	99 (94 to 105)
		Rate	531 (509.2 to 554.3)	500.6 (476.1 to 527.5)	− 6 (− 8 to − 4)	358.8 (337.7 to 382.5)	354.5 (332.2 to 379.2)	− 1 (− 3 to 0)	158.7 (149.4 to 168.8)	133.2 (119.5 to 146.9)	− 16 (− 22 to − 10)	58.9 (55 to 63.2)	58 (53.3 to 62.7)	− 2 (− 4 to 1)
World Bank lower middle income	Age-standardised	Rate	1075.4 (1026.1 to 1131.5)	1076.8 (1020.1 to 1142.5)	0 (− 1 to 2)	789.6 (741.2 to 845.9)	823.1 (767.8 to 887.4)	4 (3 to 6)	241.7 (224.3 to 259.8)	214.4 (192.9 to 236.7)	− 11 (− 16 to − 7)	103 (94.8 to 111.8)	97.2 (88.9 to 106.8)	− 6 (−7 to − 4)
		Female to male ratio	1.11	1.01	− 9	1.09	0.97	− 11	1.10	1.06	− 3	1.37	1.34	− 2
	All ages	Number (million)	12.5 (11.9 to 13.1)	25.7 (24.3 to 27.2)	106 (103 to 109)	8.8 (8.3 to 9.4)	19 (17.7 to 20.5)	116 (112 to 119)	3.3 (3 to 3.5)	5.7 (5.1 to 6.3)	75 (66 to 84)	1.3 (1.2 to 1.5)	2.6 (2.3 to 2.8)	91 (87 to 95)
		Rate	646.7 (617.9 to 679.8)	823.3 (780 to 872.6)	27 (25 to 29)	457.5 (429.5 to 489)	610.2 (568.8 to 656.8)	33 (31 to 36)	168.7 (157 to 181.5)	182.5 (164 to 201.2)	8 (3 to 14)	69.8 (64.4 to 75.6)	82.3 (74.8 to 90.5)	18 (15 to 20)
World Bank upper middle income	Age-standardised	Rate	1397.7 (1334.7 to 1466)	1553.2 (1459.9 to 1648.5)	11 (8 to 14)	1017.6 (956.2 to 1082.3)	1277.1 (1186.7 to 1375.5)	25 (22 to 29)	337.1 (312.8 to 362.4)	264.6 (239.5 to 290.9)	− 22 (− 25 to − 18)	107.8 (99.4 to 117.7)	97.3 (88.9 to 107.3)	− 10 (− 12 to − 8)
		Female to male ratio	1.04	0.94	− 10	1.00	0.91	− 9	1.08	0.97	− 10	1.38	1.35	− 2
	All ages	Number (million)	23 (22 to 24.1)	50.7 (47.6 to 53.8)	121 (114 to 127)	16.1 (15.2 to 17.2)	41.2 (38.2 to 44.5)	155 (147 to 163)	6.1 (5.7 to 6.6)	8.7 (7.9 to 9.6)	43 (36 to 51)	1.9 (1.7 to 2.1)	3.2 (2.9 to 3.6)	71 (67 to 76)
		Rate	1087.7 (1041.3 to 1140.7)	1922.9 (1806.4 to 2042.6)	77 (72 to 82)	763.9 (718.4 to 812.8)	1563.5 (1450.8 to 1688.1)	105 (98 to 111)	288.6 (267.9 to 310.9)	330.5 (298.3 to 364.6)	15 (9 to 21)	89.6 (82.6 to 97.8)	123 (111.8 to 136.1)	37 (34 to 41)
World Bank high income	Age-standardised	Rate	1258.4 (1208.4 to 1312.2)	1163.2 (1108.7 to 1221.2)	− 8 (− 10 to − 5)	996 (947.2 to 1048.5)	911.7 (858.3 to 970.4)	− 8 (− 12 to − 5)	165.3 (152.9 to 178)	143.5 (130.6 to 156.8)	− 13 (− 17 to − 9)	164 (152.7 to 176.8)	171.8 (157.8 to 187.5)	5 (3 to 7)
		Female to male ratio	0.91	0.94	3	0.82	0.83	2	1.05	1.07	2	1.62	1.69	4
	All ages	Number (million)	15.9 (15.2 to 16.5)	23.9 (22.6 to 25.1)	51 (46 to 55)	12.7 (12 to 13.4)	19.3 (18.1 to 20.5)	53 (47 to 58)	2 (1.8 to 2.1)	2.5 (2.3 to 2.7)	27 (21 to 32)	2 (1.8 to 2.1)	3.1 (2.8 to 3.4)	57 (54 to 61)
		Rate	1584.4 (1520 to 1653.6)	2006 (1901.4 to 2109.8)	27 (23 to 30)	1265.2 (1200.2 to 1336.2)	1623.1 (1517.4 to 1727.4)	28 (23 to 33)	198.3 (183.4 to 213.5)	211 (192.2 to 230.4)	6 (2 to 11)	197 (183.1 to 212.8)	260.3 (239.2 to 284.8)	32 (29 to 35)
Deaths														
Global	Age-standardised	Rate	120.8 (118.4 to 125.3)	80.5 (78.9 to 82.6)	− 33 (− 35 to − 32)	55.9 (53.9 to 57.9)	36.6 (35.5 to 38)	− 34 (− 36 to − 32)	54.3 (51.4 to 58.6)	38.2 (37 to 39.4)	− 30 (− 33 to − 27)	10.7 (8.4 to 12.2)	5.7 (5.3 to 6.3)	− 47 (− 52 to − 34)
		Female to male ratio	0.86	0.75	− 13	0.93	0.80	− 14	0.77	0.69	− 10	0.98	0.85	− 13
	All ages	Number (million)	4.4 (4.3 to 4.5)	6.2 (6 to 6.3)	41 (38 to 45)	1.9 (1.8 to 1.9)	2.7 (2.7 to 2.9)	47 (43 to 52)	2.1 (2 to 2.2)	3 (2.9 to 3.1)	44 (36 to 50)	0.4 (0.3 to 0.5)	0.4 (0.4 to 0.5)	4 (− 6 to 29)
		Rate	80.9 (79.2 to 83.8)	80.7 (79.1 to 82.8)	0 (− 3 to 2)	34.6 (33.3 to 35.9)	36 (34.8 to 37.4)	4 (1 to 7)	38.3 (36.2 to 41.4)	38.9 (37.7 to 40.2)	2 (− 4 to 6)	8 (6.3 to 9.1)	5.8 (5.5 to 6.4)	− 27 (− 34 to − 9)
World Bank low income	Age-standardised	Rate	143.1 (132.1 to 153.9)	106.7 (98.6 to 114.8)	− 25 (− 30 to − 20)	55.9 (48.6 to 63.4)	43.1 (36.3 to 48.9)	− 23 (− 28 to − 17)	79.5 (70.8 to 88.6)	58.5 (52.9 to 63.8)	− 26 (− 32 to − 20)	7.7 (5.4 to 10.9)	5.1 (3.7 to 7.7)	− 34 (− 42 to − 23)
		Female to male ratio	1.01	0.92	− 9	1.30	1.14	− 13	0.88	0.81	− 8	0.72	0.65	− 10
	All ages	Number (million)	0.2 (0.2 to 0.2)	0.3 (0.2 to 0.3)	48 (36 to 61)	0.1 (0.1 to 0.1)	0.1 (0.1 to 0.1)	59 (45 to 73)	0.1 (0.1 to 0.1)	0.2 (0.1 to 0.2)	44 (32 to 58)	0 (0 to 0)	0 (0 to 0)	30 (12 to 51)
		Rate	54.7 (50.4 to 59.1)	40 (37.3 to 42.6)	− 27 (− 33 to − 21)	17.9 (15.5 to 20.5)	14.1 (12 to 16)	− 21 (− 28 to − 14)	33 (29 to 37.4)	23.5 (21.4 to 25.6)	− 29 (− 35 to − 22)	3.8 (2.7 to 5.1)	2.4 (1.7 to 3.6)	− 36 (− 44 to − 25)
World Bank lower middle income	Age-standardised	Rate	133.6 (128 to 140.2)	102.6 (98.8 to 106.6)	− 23 (− 27 to − 20)	64 (59 to 70.4)	47.6 (43.7 to 52.7)	− 26 (− 29 to − 21)	60.7 (53.5 to 66.3)	48.2 (43.9 to 51.3)	− 21 (− 26 to − 15)	8.9 (7.4 to 11.3)	6.8 (6 to 8)	− 24 (− 32 to − 14)
		Female to male ratio	0.96	0.85	− 12	1.11	0.91	− 18	0.82	0.79	− 5	0.95	0.90	− 5
	All ages	Number (million)	1.2 (1.1 to 1.2)	2 (1.9 to 2)	70 (62 to 78)	0.5 (0.4 to 0.5)	0.8 (0.7 to 0.9)	72 (63 to 82)	0.6 (0.5 to 0.6)	1 (0.9 to 1.1)	71 (60 to 83)	0.1 (0.1 to 0.1)	0.2 (0.1 to 0.2)	56 (41 to 76)
		Rate	60.1 (57.5 to 62.6)	63.3 (61 to 65.7)	5 (0 to 10)	24.7 (22.7 to 27.4)	26.3 (23.9 to 29.4)	7 (1 to 13)	30.3 (26.8 to 32.8)	32 (29.4 to 34)	6 (− 1 to 13)	5.1 (4.2 to 6.4)	4.9 (4.4 to 5.9)	− 4 (− 13 to 9)
World Bank upper middle income	Age-standardised	Rate	159.7 (155.8 to 168.1)	101.5 (99.1 to 104.3)	− 36 (− 39 to − 34)	67.9 (65.4 to 71.9)	46.7 (45.6 to 47.9)	− 31 (− 35 to − 28)	75.1 (71.5 to 84.4)	48.5 (46.9 to 50.2)	− 35 (− 42 to − 32)	16.7 (10.8 to 19.1)	6.3 (5.6 to 6.8)	− 62 (− 68 to − 45)
		Female to male ratio	0.87	0.71	− 19	0.96	0.76	− 21	0.78	0.65	− 17	0.96	0.81	− 15
	All ages	Number (million)	2.1 (2 to 2.2)	3.1 (3 to 3.1)	48 (42 to 53)	0.8 (0.8 to 0.8)	1.4 (1.3 to 1.4)	72 (63 to 81)	1 (1 to 1.1)	1.5 (1.4 to 1.5)	46 (32 to 55)	0.2 (0.2 to 0.3)	0.2 (0.2 to 0.2)	− 20 (− 32 to 15)
		Rate	97.6 (95.3 to 102.5)	116 (113.2 to 119.2)	19 (14 to 23)	37.7 (36.3 to 40.1)	51.9 (50.6 to 53.2)	38 (30 to 45)	48.4 (46.1 to 54.4)	56.7 (54.9 to 58.8)	17 (6 to 24)	11.5 (7.6 to 13.1)	7.4 (6.5 to 8)	− 36 (− 45 to − 8)
World Bank high income	Age-standardised	Rate	71 (70.2 to 72)	31.4 (30.7 to 32.7)	− 56 (− 57 to − 54)	39.6 (39.2 to 40.2)	15.9 (15.5 to 16.6)	− 60 (− 61 to − 58)	25.7 (25.3 to 26.1)	12.1 (11.8 to 12.5)	− 53 (− 54 to − 51)	5.7 (5.5 to 5.9)	3.4 (3.3 to 3.7)	− 39 (− 41 to − 37)
		Female to male ratio	0.79	0.76	− 4	0.79	0.78	− 2	0.72	0.69	− 4	1.18	0.97	− 17
	All ages	Number (million)	0.9 (0.9 to 1)	0.8 (0.8 to 0.9)	− 12 (− 14 to − 8)	0.5 (0.5 to 0.5)	0.4 (0.4 to 0.5)	− 15 (− 17 to − 11)	0.3 (0.3 to 0.3)	0.3 (0.3 to 0.3)	− 11 (− 14 to − 7)	0.1 (0.1 to 0.1)	0.1 (0.1 to 0.1)	8 (4 to 12)
		Rate	93.8 (92.7 to 95.1)	69.7 (68.2 to 72.7)	− 26 (− 27 to − 23)	52.6 (52 to 53.4)	37.7 (36.8 to 39.5)	− 28 (− 30 to − 25)	34 (33.5 to 34.6)	25.5 (24.8 to 26.6)	− 25 (− 27 to − 22)	7.2 (7 to 7.6)	6.5 (6.3 to 6.9)	− 9 (− 12 to − 6)
DALYs (disability-adjusted life years)														
Global	Age-standardised	Rate	2392.7 (2316.5 to 2478.9)	1657.2 (1587.4 to 1723.8)	− 31 (− 33 to − 29)	948.8 (891.4 to 1008.2)	702.8 (649.5 to 756.5)	− 26 (− 29 to − 23)	1158.4 (1095.2 to 1242.3)	800.3 (773.3 to 826.1)	− 31 (− 34 to − 28)	285.5 (235.2 to 321.9)	154.1 (143.7 to 170.1)	− 46 (− 51 to − 35)
		Female to male ratio	0.84	0.73	− 13	0.90	0.79	− 12	0.76	0.66	− 13	1.00	0.89	−11
	All ages	Number (million)	98.9 (95.6 to 102.5)	132.1 (126.5 to 137.4)	34 (29 to 37)	36.6 (34.3 to 39)	55.1 (50.9 to 59.4)	50 (45 to 56)	49.3 (46.6 to 52.8)	64.5 (62.3 to 66.6)	31 (24 to 36)	12.9 (10.7 to 14.5)	12.4 (11.6 to 13.7)	− 4 (− 12 to 15)
		Rate	1832.8 (1772.6 to 1900.2)	1728.3 (1655.6 to 1797.7)	− 6 (− 9 to − 3)	679.3 (635.7 to 723.2)	721.7 (666.6 to 777.7)	6 (2 to 10)	914.1 (863 to 977.8)	844.3 (815.8 to 871.4)	− 8 (− 12 to − 4)	239.5 (197.6 to 268.6)	162.3 (151.3 to 179.1)	− 32 (− 38 to − 19)
World Bank low income	Age-standardised	Rate	2954.9 (2713.1 to 3182.4)	2153.7 (2005.6 to 2280)	− 27 (− 33 to − 21)	975.2 (855.3 to 1104.4)	761.5 (661.4 to 857.8)	− 22 (− 28 to − 16)	1758.7 (1554.7 to 1993.4)	1245.5 (1137.3 to 1354.8)	− 29 (− 35 to − 22)	221.1 (160.9 to 294.1)	146.6 (110.1 to 209)	− 34 (− 42 to − 23)
		Female to male ratio	1.01	0.88	− 12	1.36	1.14	− 16	0.89	0.78	− 12	0.74	0.69	− 7
	All ages	Number (million)	4.9 (4.4 to 5.3)	6.8 (6.4 to 7.2)	40 (28 to 54)	1.4 (1.2 to 1.6)	2.1 (1.9 to 2.4)	54 (41 to 67)	3 (2.6 to 3.5)	4.1 (3.8 to 4.5)	36 (23 to 52)	0.5 (0.4 to 0.6)	0.6 (0.4 to 0.8)	23 (8 to 46)
		Rate	1483.1 (1348.5 to 1622.4)	1027.2 (962.8 to 1080.8)	− 31 (− 37 to − 24)	418.1 (365.6 to 476.2)	318.2 (278 to 359.5)	− 24 (− 30 to − 17)	918.2 (794.7 to 1059.9)	619.5 (564.8 to 676.4)	− 33 (− 39 to − 25)	146.8 (106.8 to 184.6)	89.5 (67.2 to 123.7)	− 39 (− 47 to − 28)
World Bank lower middle income	Age-standardised	Rate	2635.2 (2526.1 to 2743.2)	2046.2 (1968.4 to 2122.8)	− 22 (− 26 to − 19)	1058 (968.4 to 1168.8)	822.3 (748 to 918.8)	− 22 (− 26 to − 18)	1322.2 (1180.6 to 1429.9)	1034 (956.2 to 1095)	− 22 (− 27 to − 17)	255.1 (213.5 to 311)	189.9 (169.5 to 223.6)	− 26 (− 32 to − 17)
		Female to male ratio	0.93	0.82	− 12	1.08	0.89	− 18	0.82	0.75	− 8	1.00	0.92	− 7
	All ages	Number (million)	29.2 (27.8 to 30.4)	47.1 (45.2 to 48.8)	61 (52 to 69)	10.1 (9.2 to 11.3)	17.3 (15.6 to 19.4)	70 (62 to 80)	15.5 (13.9 to 16.7)	24.9 (23.1 to 26.3)	61 (49 to 71)	3.5 (3 to 4.2)	5 (4.4 to 5.8)	40 (27 to 57)
		Rate	1511.2 (1441.5 to 1575.9)	1508.1 (1449.4 to 1565)	0 (− 6 to 5)	525.6 (478.9 to 584.3)	553 (499.6 to 621.7)	5 (− 0 to 11)	801.7 (718.3 to 865.6)	796.4 (739.8 to 841.5)	− 1 (− 8 to 6)	184 (153.6 to 218.6)	158.7 (141.3 to 186.5)	− 14 (− 21 to − 3)
World Bank upper middle income	Age-standardised	Rate	3061.9 (2961.4 to 3199.4)	1966.7 (1873.3 to 2062.6)	− 36 (− 38 to − 34)	1151.4 (1084.9 to 1225.4)	882.9 (812.8 to 952.1)	− 23 (− 28 to − 19)	1512.5 (1440.7 to 1686.6)	932 (899.2 to 967.7)	− 38 (− 44 to − 35)	398 (279.3 to 449.3)	151.8 (137.1 to 163.9)	− 62 (− 67 to − 48)
		Female to male ratio	0.84	0.67	− 20	0.90	0.74	− 18	0.76	0.59	− 22	0.97	0.82	− 16
	All ages	Number (million)	47.4 (45.8 to 49.4)	63.1 (60.1 to 66.2)	33 (28 to 38)	16.4 (15.4 to 17.5)	27.9 (25.6 to 30.1)	70 (60 to 78)	24.2 (23 to 26.9)	30.4 (29.3 to 31.6)	26 (15 to 33)	6.8 (4.9 to 7.6)	4.9 (4.4 to 5.3)	− 28 (− 37 to − 4)
		Rate	2244.3 (2168.7 to 2341.8)	2396.2 (2281.6 to 2513.2)	7 (3 to 10)	778.5 (730.5 to 829.2)	1057.8 (972.4 to 1141.2)	36 (28 to 43)	1145.8 (1090.9 to 1273)	1153.4 (1112.7 to 1198.1)	1 (− 8 to 6)	320 (230.3 to 360.1)	185 (166.4 to 200.1)	− 42 (− 50 to − 23)
World Bank high income	Age-standardised	Rate	1302.5 (1244.4 to 1356.5)	652.2 (600.1 to 703.9)	− 50 (− 52 to − 48)	638.8 (596.6 to 679.7)	321.2 (282.1 to 358.9)	− 50 (− 53 to − 47)	493 (481.6 to 503.7)	225.9 (218.3 to 234.4)	− 54 (− 55 to − 53)	170.8 (162.8 to 178.6)	105.1 (97.3 to 113.4)	− 38 (− 41 to − 35)
		Female to male ratio	0.76	0.77	1	0.75	0.77	2	0.66	0.64	− 4	1.19	1.14	− 4
	All ages	Number (million)	16.9 (16.1 to 17.5)	14.2 (13.1 to 15.2)	− 16 (− 19 to − 13)	8.5 (8 to 9.1)	7.5 (6.7 to 8.4)	− 11 (− 16 to − 7)	6.3 (6.2 to 6.4)	4.7 (4.6 to 4.9)	− 25 (− 27 to − 23)	2 (1.9 to 2.1)	1.9 (1.8 to 2.1)	− 6 (− 9 to − 1)
		Rate	1685.7 (1611.6 to 1753.7)	1191.5 (1098.4 to 1280.2)	− 29 (− 32 to − 27)	852.1 (797.6 to 905.1)	634.3 (562.7 to 702.8)	− 26 (− 30 to − 22)	630.2 (616.6 to 643.6)	395.5 (382.9 to 409.7)	− 37 (− 39 to − 35)	203.4 (193.8 to 212.8)	161.7 (149.7 to 174.6)	− 21 (− 24 to − 17)

Data were extracted from [8]. Rates are defined per 100,000 people

In contrast, stroke prevalence has increased over time from 1990 to 2017, likely because of longer survival and reduced mortality of people experiencing a stroke. In 2017, the crude number of people with a stroke was 104.2 million (UI 98.5–110.1), which has almost doubled, particularly for ischaemic stroke, compared to the number in 1990 (Table 2). The global rate of age-standardised stroke prevalence has increased by 3% (UI 1–5%) from 1990 to 2017 to reach to 1300.6 (UI 1229.0–1374.7) per 100,000 in 2017; particularly in UMICs (11%, UI 8–14%). This increase in prevalence could be partly explained by improved healthcare (including screening, prevention, diagnosis, and treatment) and general awareness, which has extended the lifespan of stroke patients in these income categories. Contrarily, both LICs and HICs have exhibited a respective 3% (UI 1–5%) and 8% (UI 5–10%) decrease in the age-standardised rates of stroke prevalent cases by 2017. Of note, in contrast to ischaemic strokes, the age-standardised rates of haemorrhagic strokes have significantly decreased worldwide from 1990 to 2017.

Stroke is the second leading cause of death worldwide, with regard to the age-standardised global stroke mortality rate (i.e. stroke-related deaths per 100,000), and this rank has remained relatively constant in different regions since 1990 (Table 1). Because of population growth, particularly among the elderly, the crude number of stroke events and mortality has dramatically increased worldwide from 1990 to 2017. However, compared to 1990, the age-standardised global (ischaemic and haemorrhagic) stroke mortality rate has decreased by 33% (UI 32–35; − 25% in LICs, − 23% in LMICs, − 36% in UMICs, and − 56% in HICs). There were 80.5 (UI 78.9–82.6) deaths per 100,000 in 2017, 45% of which were related to ischaemic strokes (Table 2).

In 2017, stroke has imposed 132.1 (126.5 to 137.4) million DALYs lost globally (34% more than in 1990), 42% of which was related to ischaemic strokes, in particular, 6.8 million DALYs in LICs, 47.1 million DALYs in LMICs, 63.1 million DALYs in UMICs, and 14.2 million DALYs in HICs (Table 2). Nevertheless, the age-standardised rate of DALYs lost has decreased globally by 31% (UI 29–33), from 2392.7 (UI 2316.5–2478.9) in 1990 to 1657.2 (1587.4–1723.8) in 2017. The decrease was more prominent in HICs and UMICs. The highest age-standardised rate of DALYs lost in 2017 for ischaemic stroke was in UMICs (882.9, UI 812.8–952.1); for intracerebral haemorrhage, the highest rate was in LICs (1245.5, UI1137.3–1354.8); and for subarachnoid haemorrhage the highest rate was in LMICs (189.9, UI 169.5–223.6). Female to male ratio of stroke-related global DALYs lost was 0.73 in 2017, which has decreased by 13% compared to that of 1990.

Overall, comparing the most-affected and the least-affected income regions based on the age-standardised rates, there is a 1.7-fold difference in stroke events (ranging from 190.1 [UI 176.6–205] per 100,000 in UMICs to 114.7 [UI 107.3–123] per 100,000 in HICs); a 1.6-fold difference in stroke prevalent cases (ranging from 1553.2 [UI 1459.9–1648.5] per 100,000 in LICs to 996.0 [UI 949.6–1049.3] per 100,000 in UMICs); a 3.4-fold difference in stroke-related deaths (ranging from 106.7 [UI 98.6–114.8] per 100,000 in LICs to 31.4 [UI 30.7–32.7] per 100,000 in HICs); and a 3.3-fold difference in stroke DALYs (ranging from 2153.7 [UI 2005.6–2280] per 100,000 in LICs to 652.2 [UI 600.1–703.9] per 100,000 in HICs).

### Modifiable predictors of stroke mortality

In 2017, 5.2 million stroke-related deaths and 116.3 million stroke-related DALYs lost worldwide were attributable to modifiable risk factors, less than half of which were observed for ischaemic strokes (Additional file 2: Table S1). Since 1990, the mean age-standardised global (ischaemic and haemorrhagic) stroke mortality rates attributable to modifiable risk factors have declined by 34% (UI 30–37%), ranging from 23% in LMICs to 58% in HICs (Table 3 and Additional file 4: Table S3). The age-standardised rate of global stroke deaths per 100,000 attributable to modifiable risk factors was 67.9 (UI 64.2–71.3) per 100,000 in 2017. This could be explained by improvements in quality of life and many SES determinants, particularly infrastructures, healthcare, and general awareness. However, some unhealthy habits of modern life have inevitably resulted in an increased stroke mortality rates attributable to some of the underlying risk factors, such as smoking, obesity, and alcohol drinking in less wealthy societies. Overall, there is a twofold to fivefold difference in stroke mortality rates attributable to modifiable risk factors between the most-affected and the least-affected SES-specific countries. Common predictors of stroke risk and mortality are discussed in the following and in Additional file 1: Text S1.

**Table 3:** Age-standardised rates of stroke mortality and burden attributable to modifiable risk factors

	All-type stroke			Ischaemic stroke			Intracerebral haemorrhage			Subarachnoid haemorrhage		
	Mean (95% uncertainty interval)			Mean (95% uncertainty interval)			Mean (95% uncertainty interval)			Mean (95% uncertainty interval)		
	1990	2017	Percent change	1990	2017	Percent change	1990	2017	Percent change	1990	2017	Percent change
Deaths per 100,000 people												
Global												
All risk factors	102.7 (97.5 to 108.4)	67.9 (64.2 to 71.3)	-34 (-36 to -32)	46.7 (42.4 to 50.9)	30.2 (27.4 to 33)	-35 (-37 to -33)	47.2 (43.9 to 51.6)	33 (30.9 to 35.1)	-30 (-34 to -27)	8.9 (7 to 10.3)	4.7 (4.3 to 5.3)	-47 (-52 to -34)
Air pollution	14.5 (12 to 17)	8.6 (7 to 10.3)	-41 (-43 to -38)	5.6 (4.5 to 6.7)	3.4 (2.7 to 4.2)	-38 (-41 to -35)	7.4 (6.1 to 8.8)	4.5 (3.6 to 5.4)	-39 (-44 to -36)	1.5 (1.1 to 1.9)	0.7 (0.5 to 0.8)	-56 (-61 to -43)
Other environmental risks	6.1 (4 to 8.5)	4.7 (3.2 to 6.5)	-22 (-27 to -16)	2 (1.2 to 3.1)	1.8 (1.1 to 2.6)	*-11 (-19 to 1)*	3.4 (2.2 to 4.9)	2.6 (1.7 to 3.7)	-23 (-30 to -16)	0.7 (0.4 to 1)	0.3 (0.2 to 0.5)	-53 (-60 to -36)
Tobacco	22.5 (21.1 to 24.1)	13.2 (12.3 to 14.1)	-42 (-44 to -39)	8.7 (8 to 9.4)	5.1 (4.7 to 5.5)	-41 (-44 to -39)	11.4 (10.5 to 12.6)	7 (6.5 to 7.5)	-38 (-42 to -35)	2.4 (1.9 to 2.9)	1 (0.9 to 1.2)	-57 (-62 to -46)
Alcohol use	8.2 (5.2 to 11.2)	7.1 (5.1 to 9.1)	*-13 (-29 to 12)*	*0.9 (-0.8 to 2.9)*	1.3 (0.3 to 2.3)	*34 (-1079 to 841)*	7.2 (5 to 9.4)	5.8 (4.2 to 7.4)	-20 (-31 to -3)	0 (0 to 0)	0 (0 to 0)	0 (0 to 0)
High fasting plasma glucose	22 (13.9 to 36.2)	16.5 (10.9 to 25.6)	-25 (-30 to -20)	11.1 (5.3 to 24.1)	7.9 (3.9 to 16.5)	-29 (-34 to -23)	9.3 (5.7 to 14.5)	7.5 (4.6 to 11.4)	-19 (-25 to -14)	1.6 (1 to 2.5)	1.1 (0.7 to 1.7)	-31 (-38 to -16)
High systolic blood pressure	64.4 (54.2 to 73.8)	43.4 (36.6 to 49.9)	-33 (-35 to -31)	28 (21.6 to 34.6)	18.1 (13.9 to 22.3)	-36 (-38 to -33)	30.5 (24.1 to 37)	22.1 (17.5 to 26.4)	-28 (-32 to -24)	5.9 (4.4 to 7.2)	3.3 (2.6 to 3.9)	-44 (-50 to -32)
High body-mass index	13.7 (7 to 21.8)	13.2 (8 to 18.9)	*-4 (-15 to 16)*	4.7 (2.3 to 7.8)	4.1 (2.3 to 6.3)	-13 (-21 to -1)	7.3 (3.7 to 12.1)	7.6 (4.7 to 11.2)	*5 (-10 to 28)*	1.7 (0.9 to 2.7)	1.5 (1 to 2)	*-12 (-29 to 14)*
Dietary risks	58.7 (52.4 to 65)	37.1 (32.8 to 41.3)	-37 (-39 to -35)	22.7 (18.8 to 26.5)	14.4 (12 to 16.8)	-36 (-39 to -33)	29.8 (25.1 to 34.8)	19.7 (16.7 to 22.5)	-34 (-38 to -30)	6.2 (4.6 to 7.4)	3 (2.4 to 3.5)	-52 (-58 to -39)
Low physical activity	6.3 (2.2 to 11.6)	4 (1.4 to 7.3)	-37 (-40 to -34)	6.3 (2.2 to 11.6)	4 (1.4 to 7.3)	-37 (-40 to -34)	0 (0 to 0)	0 (0 to 0)	0 (0 to 0)	0 (0 to 0)	0 (0 to 0)	0 (0 to 0)
Impaired kidney function	9.2 (6.9 to 11.8)	6 (4.6 to 7.6)	-35 (-38 to -31)	4.7 (3.3 to 6.3)	2.9 (2.1 to 3.8)	-38 (-41 to -34)	4.5 (3.5 to 5.5)	3.1 (2.5 to 3.7)	-31 (-35 to -27)	0 (0 to 0)	0 (0 to 0)	0 (0 to 0)
High LDL cholesterol	11.4 (4 to 23.6)	7 (2.6 to 14.5)	-38 (-41 to -34)	11.4 (4 to 23.6)	7 (2.6 to 14.5)	-38 (-41 to -34)	0 (0 to 0)	0 (0 to 0)	0 (0 to 0)	0 (0 to 0)	0 (0 to 0)	0 (0 to 0)
World Bank Low Income												
All risk factors	120.6 (109.6 to 131.4)	90.9 (82.8 to 98.3)	-25 (-30 to -19)	45.7 (38.4 to 53.6)	35.7 (29.3 to 41.6)	-22 (-28 to -16)	68.6 (60.3 to 77.1)	50.9 (45.6 to 56.6)	-26 (-31 to -19)	6.3 (4.5 to 9)	4.3 (3.1 to 6.5)	-32 (-41 to -20)
Air pollution	24.2 (20.6 to 28.1)	16.2 (13.5 to 19.2)	-33 (-38 to -27)	8.7 (7 to 10.5)	5.9 (4.7 to 7.3)	-31 (-37 to -25)	14.2 (11.7 to 16.8)	9.4 (7.8 to 11.2)	-33 (-39 to -27)	1.4 (0.9 to 2)	0.9 (0.6 to 1.3)	-39 (-47 to -29)
Other environmental risks	9.4 (6.2 to 12.9)	7.1 (4.7 to 9.8)	-25 (-31 to -17)	3.1 (1.9 to 4.5)	2.6 (1.6 to 3.8)	-16 (-23 to -7)	5.7 (3.6 to 8.2)	4.1 (2.6 to 5.8)	-28 (-35 to -20)	0.6 (0.3 to 0.9)	0.4 (0.2 to 0.6)	-34 (-43 to -22)
Tobacco	15.4 (13.5 to 17.3)	10.7 (9.5 to 11.9)	-30 (-36 to -24)	4.8 (4.1 to 5.8)	3.5 (2.9 to 4.2)	-27 (-34 to -20)	9.5 (8.2 to 10.9)	6.5 (5.7 to 7.3)	-31 (-37 to -24)	1.1 (0.8 to 1.6)	0.7 (0.5 to 1.1)	-38 (-48 to -25)
Alcohol use	7.1 (3.9 to 10.7)	4.1 (1.6 to 7)	-42 (-63 to -27)	0 (-1.1 to 1.1)	*-0.4 (-1.4 to 0.5)*	*4097 (-1508 to 1024)*	7.1 (4.3 to 10.2)	4.5 (2.2 to 6.9)	-36 (-51 to -24)	0 (0 to 0)	0 (0 to 0)	0 (0 to 0)
High fasting plasma glucose	32.6 (20.8 to 50.7)	27.1 (17.1 to 41.8)	-17 (-24 to -9)	13.3 (6 to 28.8)	11.4 (5.2 to 25)	-14 (-22 to -5)	17.9 (9.9 to 28.9)	14.5 (8.3 to 22.9)	-19 (-27 to -10)	1.5 (0.8 to 2.7)	1.2 (0.6 to 2.1)	-22 (-33 to -8)
High systolic blood pressure	73.5 (60.6 to 86)	59.1 (49.2 to 68.1)	-20 (-25 to -14)	26.2 (19.7 to 33.7)	21.5 (16.1 to 27.5)	-18 (-24 to -11)	43.3 (33.5 to 53.2)	34.6 (27.4 to 41.3)	-20 (-26 to -13)	4.1 (2.8 to 6)	3 (2 to 4.6)	-28 (-37 to -16)
High body-mass index	11 (4.2 to 20.8)	12.9 (6.9 to 20.1)	*17 (-5 to 67)*	2.7 (1 to 5.2)	3.3 (1.7 to 5.5)	24 (0 to 76)	7.5 (2.9 to 14.2)	8.7 (4.6 to 13.8)	*16 (-6 to 65)*	0.9 (0.3 to 1.7)	0.9 (0.4 to 1.6)	*6 (-17 to 59)*
Dietary risks	69 (59.2 to 79.2)	49.2 (42.2 to 56.6)	-29 (-34 to -23)	22.9 (17.7 to 28.3)	17.2 (13.2 to 21.3)	-25 (-31 to -19)	41.9 (33.8 to 50.7)	29.4 (23.9 to 35.4)	-30 (-36 to -24)	4.2 (3 to 6.1)	2.7 (1.9 to 4)	-36 (-45 to -26)
Low physical activity	5.5 (1.8 to 10.5)	4.2 (1.5 to 8.1)	-22 (-29 to -16)	5.5 (1.8 to 10.5)	4.2 (1.5 to 8.1)	-22 (-29 to -16)	0 (0 to 0)	0 (0 to 0)	0 (0 to 0)	0 (0 to 0)	0 (0 to 0)	0 (0 to 0)
Impaired kidney function	12.5 (9.4 to 15.6)	9.3 (7 to 11.8)	-26 (-32 to -20)	5.2 (3.6 to 7)	4 (2.7 to 5.4)	-24 (-31 to -18)	7.3 (5.6 to 9.1)	5.3 (4.2 to 6.6)	-27 (-33 to -20)	0 (0 to 0)	0 (0 to 0)	0 (0 to 0)
High LDL cholesterol	8.4 (3.3 to 18.5)	7 (2.7 to 15.2)	-16 (-23 to -9)	8.4 (3.3 to 18.5)	7 (2.7 to 15.2)	-16 (-23 to -9)	0 (0 to 0)	0 (0 to 0)	0 (0 to 0)	0 (0 to 0)	0 (0 to 0)	0 (0 to 0)
World Bank Lower Middle Income												
All risk factors	113.6 (106.8 to 121.1)	87.7 (82.5 to 92.7)	-23 (-26 to -20)	53.2 (46.9 to 60.3)	39.7 (35 to 44.9)	-25 (-29 to -21)	52.9 (46.7 to 58.7)	42.2 (38.3 to 45.7)	-20 (-25 to -15)	7.5 (6.2 to 9.7)	5.8 (5 to 6.9)	-23 (-32 to -13)
Air pollution	19.1 (16.1 to 22.2)	12.3 (10.1 to 14.7)	-36 (-40 to -31)	8 (6.4 to 9.6)	5.1 (4 to 6.3)	-36 (-41 to -31)	9.7 (7.8 to 11.4)	6.3 (5.1 to 7.5)	-35 (-40 to -30)	1.5 (1.1 to 2)	1 (0.7 to 1.2)	-36 (-43 to -27)
Other environmental risks	7.7 (5.1 to 10.5)	6.7 (4.5 to 9.1)	-13 (-18 to -6)	2.8 (1.7 to 4.2)	2.7 (1.7 to 4)	*-3 (-12 to 8)*	4.2 (2.7 to 6)	3.5 (2.3 to 4.9)	-17 (-24 to -10)	0.6 (0.4 to 1)	0.5 (0.3 to 0.7)	-23 (-34 to -7)
Tobacco	20.5 (18.7 to 22.5)	13.8 (12.6 to 15.1)	-33 (-38 to -28)	8.2 (7.2 to 9.4)	5.3 (4.7 to 6.1)	-35 (-40 to -30)	10.7 (9.3 to 12.1)	7.4 (6.6 to 8.2)	-31 (-37 to -25)	1.6 (1.2 to 2.3)	1.1 (0.9 to 1.4)	-34 (-44 to -19)
Alcohol use	4.9 (2.6 to 7.3)	6.6 (4.9 to 8.5)	34 (2 to 101)	0.6 (-0.7 to 1.9)	1.5 (0.7 to 2.3)	*159 (-2053 to 2162)*	4.4 (2.9 to 5.9)	5.1 (3.7 to 6.7)	*17 (-4 to 52)*	0 (0 to 0)	0 (0 to 0)	0 (0 to 0)
High fasting plasma glucose	27.1 (16.8 to 45.8)	25.9 (16.9 to 40.7)	*-4 (-13 to 5)*	13.6 (6.2 to 30.9)	12.3 (5.8 to 25.9)	*-9 (-18 to 2)*	11.9 (6.8 to 19.2)	12 (7.2 to 18.3)	*1 (-8 to 11)*	1.6 (1 to 2.6)	1.6 (1 to 2.4)	*-1 (-15 to 14)*
High systolic blood pressure	71.4 (60.7 to 81.9)	57 (48.7 to 65.4)	-20 (-24 to -17)	31.6 (24.4 to 40)	24.1 (18.7 to 30.7)	-24 (-28 to -19)	34.8 (27 to 42.2)	28.9 (23 to 34.6)	-17 (-23 to -11)	5.1 (3.9 to 6.7)	4.1 (3.2 to 5.1)	-20 (-28 to -10)
High body-mass index	11.1 (5.5 to 18.4)	14.7 (9 to 21)	33 (10 to 69)	3.9 (1.9 to 6.6)	4.3 (2.5 to 6.6)	*11 (-3 to 34)*	6.1 (2.9 to 10.5)	8.8 (5.3 to 12.7)	45 (17 to 92)	1.1 (0.5 to 1.9)	1.6 (1 to 2.3)	44 (15 to 95)
Dietary risks	63.7 (56.1 to 71.8)	44.5 (38.7 to 50.2)	-30 (-34 to -27)	26.2 (20.7 to 31.4)	17.5 (14.1 to 21.1)	-33 (-37 to -29)	32.5 (26.5 to 38.5)	23.4 (19.2 to 27.7)	-28 (-33 to -23)	5 (3.9 to 6.6)	3.5 (2.8 to 4.4)	-30 (-37 to -21)
Low physical activity	6.7 (2.2 to 12.6)	4.9 (1.7 to 9.1)	-27 (-31 to -23)	6.7 (2.2 to 12.6)	4.9 (1.7 to 9.1)	-27 (-31 to -23)	0 (0 to 0)	0 (0 to 0)	0 (0 to 0)	0 (0 to 0)	0 (0 to 0)	0 (0 to 0)
Impaired kidney function	11.3 (8.6 to 14.3)	8.9 (6.8 to 11.2)	-22 (-26 to -17)	5.8 (4.1 to 7.7)	4.3 (3 to 5.7)	-25 (-30 to -20)	5.6 (4.4 to 6.9)	4.6 (3.7 to 5.6)	-17 (-23 to -12)	0 (0 to 0)	0 (0 to 0)	0 (0 to 0)
High LDL cholesterol	11.1 (4 to 24.6)	8.2 (3.1 to 17.8)	-26 (-30 to -21)	11.1 (4 to 24.6)	8.2 (3.1 to 17.8)	-26 (-30 to -21)	0 (0 to 0)	0 (0 to 0)	0 (0 to 0)	0 (0 to 0)	0 (0 to 0)	0 (0 to 0)
World Bank Upper Middle Income												
All risk factors	133.9 (126.4 to 142.4)	84.8 (79.9 to 89.6)	-37 (-39 to -34)	56.1 (51.3 to 61.7)	38.3 (35.1 to 41.7)	-32 (-35 to -28)	64 (59 to 72.4)	41.3 (38.4 to 44.2)	-35 (-42 to -31)	13.7 (8.8 to 16)	5.2 (4.5 to 5.8)	-62 (-68 to -45)
Air pollution	19.3 (15.9 to 23)	10.2 (8 to 12.3)	-47 (-51 to -44)	6.9 (5.5 to 8.4)	4.2 (3.3 to 5.2)	-39 (-44 to -34)	10 (8.2 to 12.2)	5.3 (4.2 to 6.3)	-47 (-53 to -43)	2.4 (1.5 to 3)	0.7 (0.5 to 0.8)	-72 (-77 to -58)
Other environmental risks	8.7 (5.7 to 12)	6.3 (4.2 to 8.7)	-27 (-33 to -20)	2.5 (1.5 to 3.8)	2.4 (1.5 to 3.5)	*-6 (-18 to 9)*	4.9 (3.1 to 7.1)	3.5 (2.3 to 5.1)	-28 (-38 to -19)	1.2 (0.6 to 1.8)	0.4 (0.2 to 0.6)	-68 (-75 to -48)
Tobacco	30.8 (28.5 to 33.5)	18.9 (17.6 to 20.3)	-39 (-43 to -35)	10.9 (10 to 12.1)	7.5 (7 to 8.1)	-31 (-36 to -25)	16.3 (14.7 to 18.5)	10.1 (9.4 to 10.8)	-38 (-44 to -33)	3.6 (2.4 to 4.4)	1.3 (1.1 to 1.5)	-65 (-72 to -49)
Alcohol use	10.7 (6.7 to 14.8)	11.1 (7.5 to 14.5)	*4 (-20 to 46)*	*0.9 (-1 to 2.9)*	2.4 (0.9 to 4)	*175 (-2437 to 2863)*	9.8 (6.7 to 13)	8.6 (6.1 to 11.3)	*-12 (-30 to 18)*	0 (0 to 0)	0 (0 to 0)	0 (0 to 0)
High fasting plasma glucose	24.3 (15.7 to 38.7)	16.2 (10.5 to 25.2)	-34 (-38 to -29)	11.6 (5.7 to 24.3)	8 (4.1 to 16.1)	-31 (-36 to -25)	10.6 (6.6 to 16.3)	7.2 (4.5 to 10.9)	-32 (-38 to -26)	2.2 (1.2 to 3.4)	1 (0.6 to 1.5)	-54 (-61 to -36)
High systolic blood pressure	81.5 (67.6 to 94.8)	53.9 (45 to 62.4)	-34 (-37 to -31)	32.8 (25.2 to 40.8)	22.9 (17.7 to 28.4)	-30 (-34 to -27)	40 (30.8 to 49.4)	27.4 (21.3 to 33.3)	-32 (-38 to -27)	8.7 (5.5 to 11.1)	3.6 (2.8 to 4.3)	-59 (-65 to -40)
High body-mass index	17.7 (8.7 to 29.1)	16.1 (9.4 to 23.7)	*-9 (-21 to 11)*	6 (2.9 to 9.9)	5.4 (2.9 to 8.4)	*-11 (-19 to 1)*	9.5 (4.5 to 16)	9.2 (5.4 to 13.9)	*-3 (-18 to 20)*	2.2 (1 to 3.8)	1.6 (1 to 2.2)	*-27 (-46 to 6)*
Dietary risks	84 (74.4 to 93.5)	48.9 (42.9 to 54.6)	-42 (-45 to -39)	30.2 (25.2 to 35.3)	19.5 (16.3 to 22.5)	-36 (-40 to -31)	43.6 (36.9 to 51.3)	26.1 (22.2 to 30)	-40 (-47 to -36)	10.2 (6.3 to 12.3)	3.3 (2.8 to 3.9)	-67 (-72 to -51)
Low physical activity	7.7 (2.6 to 14.3)	5.1 (1.8 to 9.4)	-34 (-37 to -30)	7.7 (2.6 to 14.3)	5.1 (1.8 to 9.4)	-34 (-37 to -30)	0 (0 to 0)	0 (0 to 0)	0 (0 to 0)	0 (0 to 0)	0 (0 to 0)	0 (0 to 0)
Impaired kidney function	11.4 (8.5 to 14.6)	6.8 (5.1 to 8.6)	-40 (-45 to -37)	5.6 (3.9 to 7.4)	3.4 (2.4 to 4.5)	-40 (-43 to -36)	5.7 (4.5 to 7.2)	3.4 (2.7 to 4.2)	-41 (-47 to -37)	0 (0 to 0)	0 (0 to 0)	0 (0 to 0)
High LDL cholesterol	13.7 (5 to 28.3)	9.2 (3.3 to 18.8)	-33 (-37 to -30)	13.7 (5 to 28.3)	9.2 (3.3 to 18.8)	-33 (-37 to -30)	0 (0 to 0)	0 (0 to 0)	0 (0 to 0)	0 (0 to 0)	0 (0 to 0)	0 (0 to 0)
World Bank High Income												
All risk factors	60.9 (57.2 to 64.2)	25.5 (23.6 to 27.5)	-58 (-59 to -56)	33.2 (30.1 to 36.4)	12.5 (11 to 14.2)	-62 (-64 to -60)	22.7 (21.4 to 23.9)	10.2 (9.4 to 10.9)	-55 (-57 to -53)	4.9 (4.6 to 5.2)	2.8 (2.6 to 3.1)	-42 (-45 to -39)
Air pollution	5 (3.8 to 6.3)	1.9 (1.4 to 2.4)	-62 (-65 to -60)	2.5 (1.9 to 3.3)	0.9 (0.6 to 1.1)	-66 (-68 to -64)	2 (1.5 to 2.5)	0.8 (0.6 to 1)	-61 (-64 to -58)	0.5 (0.4 to 0.6)	0.3 (0.2 to 0.3)	-49 (-53 to -46)
Other environmental risks	1.9 (0.9 to 3.1)	0.8 (0.3 to 1.3)	-60 (-64 to -57)	0.9 (0.4 to 1.6)	0.3 (0.1 to 0.6)	-62 (-66 to -60)	0.8 (0.4 to 1.4)	0.3 (0.1 to 0.6)	-59 (-65 to -55)	0.2 (0.1 to 0.3)	0.1 (0 to 0.1)	-53 (-61 to -46)
Tobacco	15.1 (14.2 to 16)	4.2 (3.9 to 4.5)	-72 (-74 to -71)	6.9 (6.4 to 7.3)	1.6 (1.5 to 1.8)	-76 (-77 to -75)	6.5 (6.1 to 6.9)	1.9 (1.7 to 2)	-71 (-73 to -69)	1.8 (1.7 to 1.9)	0.7 (0.6 to 0.7)	-63 (-65 to -60)
Alcohol use	7.1 (4.1 to 10.1)	2.1 (0.8 to 3.4)	-71 (-83 to -62)	1.2 (-1 to 3.4)	-0.1 (-0.9 to 0.8)	*-107 (-474 to 322)*	5.9 (4 to 7.6)	2.1 (1.3 to 3)	-63 (-71 to -58)	0 (0 to 0)	0 (0 to 0)	0 (0 to 0)
High fasting plasma glucose	15.1 (9 to 26.7)	7.8 (4.8 to 13.4)	-48 (-53 to -42)	8.9 (4 to 20.2)	4.1 (1.9 to 9.6)	-54 (-57 to -49)	5.2 (3.1 to 8.7)	3 (1.8 to 5)	-43 (-48 to -38)	1 (0.6 to 1.4)	0.7 (0.5 to 1.1)	-23 (-30 to -15)
High systolic blood pressure	40.1 (33.7 to 46.4)	15.4 (12.6 to 18.2)	-62 (-64 to -60)	20.8 (15.8 to 26.3)	7.1 (5.2 to 9.2)	-66 (-68 to -64)	15.8 (12.5 to 18.8)	6.4 (4.9 to 8)	-59 (-62 to -57)	3.5 (3 to 4.1)	1.9 (1.5 to 2.2)	-47 (-51 to -44)
High body-mass index	10.9 (6.3 to 16.2)	6.1 (4 to 8.4)	-44 (-50 to -35)	3.9 (2 to 6.3)	1.9 (1.1 to 3)	-52 (-56 to -44)	5.3 (3.1 to 7.9)	3.1 (2 to 4.3)	-43 (-49 to -32)	1.7 (1.1 to 2.3)	1.1 (0.8 to 1.5)	-31 (-37 to -21)
Dietary risks	26.7 (23.6 to 30)	11.4 (9.9 to 12.9)	-57 (-59 to -55)	12.9 (10.6 to 15.1)	4.9 (4 to 5.8)	-62 (-64 to -60)	11 (9.2 to 12.8)	4.9 (4.1 to 5.8)	-55 (-57 to -53)	2.8 (2.4 to 3.3)	1.6 (1.3 to 1.9)	-43 (-45 to -40)
Low physical activity	4.9 (1.6 to 9)	2 (0.6 to 3.6)	-60 (-61 to -58)	4.9 (1.6 to 9)	2 (0.6 to 3.6)	-60 (-61 to -58)	0 (0 to 0)	0 (0 to 0)	0 (0 to 0)	0 (0 to 0)	0 (0 to 0)	0 (0 to 0)
Impaired kidney function	5.2 (3.6 to 7)	2.2 (1.5 to 3)	-58 (-62 to -55)	3.2 (2 to 4.4)	1.3 (0.8 to 1.8)	-61 (-65 to -57)	2.1 (1.5 to 2.6)	0.9 (0.7 to 1.2)	-54 (-57 to -51)	0 (0 to 0)	0 (0 to 0)	0 (0 to 0)
High LDL cholesterol	9.1 (2.8 to 19.3)	3.3 (1 to 7.3)	-64 (-66 to -62)	9.1 (2.8 to 19.3)	3.3 (1 to 7.3)	-64 (-66 to -62)	0 (0 to 0)	0 (0 to 0)	0 (0 to 0)	0 (0 to 0)	0 (0 to 0)	0 (0 to 0)
DALYs (Disability-Adjusted Life Years) per 100,000 people												
Global												
All risk factors	2073.7 (1981.4 to 2169.1)	1449.6 (1378.6 to 1519.4)	-30 (-32 to -28)	819.9 (752.5 to 886)	605.6 (549.2 to 662.8)	-26 (-29 to -23)	1021.9 (960 to 1102.1)	714.4 (680.8 to 747.7)	-30 (-34 to -27)	231.9 (190 to 264.7)	129.6 (119.5 to 143.3)	-44 (-49 to -33)
Air pollution	333.9 (278.5 to 390.6)	201.9 (164.1 to 240.1)	-40 (-43 to -37)	108.9 (87.8 to 129.6)	72.2 (57.4 to 87.3)	-34 (-37 to -30)	181.9 (148.8 to 213.4)	109.7 (89.3 to 130.8)	-40 (-44 to -36)	43.1 (33 to 53.1)	19.9 (15.6 to 24.6)	-54 (-59 to -43)
Other environmental risks	142.6 (94.4 to 196.2)	101.2 (66.7 to 139.1)	-29 (-33 to -25)	42.2 (26 to 61)	37.8 (23.9 to 53.1)	-11 (-17 to -2)	81.3 (52.8 to 114.4)	54.9 (35.2 to 77.1)	-32 (-38 to -27)	19.1 (11.1 to 28.2)	8.5 (5 to 12.7)	-56 (-63 to -43)
Tobacco	559.4 (520.2 to 599.6)	340.5 (315.7 to 367.2)	-39 (-42 to -37)	196.7 (180.3 to 214)	128.7 (116.7 to 142.1)	-35 (-38 to -31)	291.2 (265.5 to 318.5)	179.1 (166.4 to 192.1)	-38 (-42 to -35)	71.6 (57.7 to 84)	32.6 (29 to 36.9)	-54 (-59 to -46)
Alcohol use	194.3 (137.3 to 252.6)	172.6 (130.4 to 214.4)	*-11 (-24 to 7)*	*27.3 (-1.4 to 58.9)*	35.1 (14.2 to 56.2)	*29 (-271 to 440)*	167 (120.6 to 211.5)	137.5 (103.1 to 171.5)	-18 (-28 to -2)	0 (0 to 0)	0 (0 to 0)	0 (0 to 0)
High fasting plasma glucose	405.5 (280.5 to 582.8)	334.9 (235.8 to 464.6)	-17 (-23 to -12)	180.8 (95.3 to 331)	150 (82.1 to 259.6)	-17 (-24 to -9)	186.6 (119.8 to 268.8)	156.9 (101.4 to 221.3)	-16 (-22 to -10)	38.1 (24.3 to 56.1)	28 (18.1 to 39.7)	-27 (-34 to -12)
High systolic blood pressure	1299.6 (1110.9 to 1465)	936.7 (805.3 to 1053)	-28 (-30 to -26)	497.6 (399.2 to 591.1)	370.8 (295.5 to 439.7)	-25 (-29 to -22)	653 (538.5 to 764.1)	478.4 (400.6 to 550.2)	-27 (-31 to -23)	149 (116.2 to 178.9)	87.4 (72.5 to 104.2)	-41 (-46 to -30)
High body-mass index	388.4 (209.5 to 604.8)	406.8 (262.6 to 557.3)	*5 (-10 to 27)*	119.1 (64.4 to 186.6)	124.3 (75.8 to 179.9)	*4 (-6 to 21)*	211.1 (110.4 to 337.6)	228.8 (146.2 to 315.5)	*8 (-8 to 35)*	58.2 (31.8 to 91.6)	53.8 (36.6 to 71.8)	*-8 (-25 to 19)*
Dietary risks	1367.2 (1228.9 to 1512)	908 (809.5 to 1004.7)	-34 (-36 to -31)	478.7 (407.9 to 550.4)	348 (293.5 to 401.4)	-27 (-31 to -23)	714.7 (614.2 to 822.3)	471.4 (406.3 to 534.8)	-34 (-38 to -31)	173.8 (136.7 to 205.7)	88.6 (75.1 to 103.3)	-49 (-54 to -38)
Low physical activity	98.9 (34.3 to 186.3)	70.1 (23.9 to 131.6)	-29 (-32 to -25)	98.9 (34.3 to 186.3)	70.1 (23.9 to 131.6)	-29 (-32 to -25)	0 (0 to 0)	0 (0 to 0)	0 (0 to 0)	0 (0 to 0)	0 (0 to 0)	0 (0 to 0)
Impaired kidney function	177.7 (145.4 to 212.9)	126.9 (104.7 to 152.2)	-29 (-32 to -25)	81.6 (63.9 to 100.1)	58.8 (47.1 to 72.3)	-28 (-32 to -23)	96.2 (79.4 to 115.1)	68 (56 to 80.5)	-29 (-33 to -26)	0 (0 to 0)	0 (0 to 0)	0 (0 to 0)
High LDL cholesterol	219.3 (122.7 to 370.8)	159.1 (93 to 269.2)	-27 (-31 to -23)	219.3 (122.7 to 370.8)	159.1 (93 to 269.2)	-27 (-31 to -23)	0 (0 to 0)	0 (0 to 0)	0 (0 to 0)	0 (0 to 0)	0 (0 to 0)	0 (0 to 0)
World Bank Low Income												
All risk factors	2555.9 (2337.6 to 2776.2)	1895.1 (1762.6 to 2011.5)	-26 (-31 to -19)	830.2 (714.9 to 954.2)	657.3 (559.3 to 749.3)	-21 (-27 to -15)	1549.7 (1363.8 to 1747.1)	1114.9 (1013.2 to 1219.3)	-28 (-34 to -21)	176.1 (128 to 239.5)	122.8 (90.4 to 177.1)	-30 (-39 to -19)
Air pollution	563.4 (469.4 to 659.7)	372.2 (310 to 434.3)	-34 (-40 to -27)	168.5 (136.2 to 205.5)	117 (93.5 to 142.3)	-31 (-37 to -24)	352.7 (286.6 to 426.1)	229 (187.9 to 271.2)	-35 (-41 to -28)	42.1 (29 to 59.8)	26.2 (18.4 to 38.9)	-38 (-46 to -27)
Other environmental risks	208.5 (136.5 to 286.1)	146.3 (96.7 to 200.1)	-30 (-36 to -21)	59.8 (37.4 to 84)	49.2 (30.9 to 68.4)	-18 (-24 to -9)	132.4 (83.3 to 188.2)	87 (54.4 to 123.2)	-34 (-41 to -26)	16.3 (9.1 to 26.2)	10.2 (5.7 to 16.4)	-38 (-46 to -24)
Tobacco	388.6 (341.7 to 437.8)	272.2 (239.1 to 303.4)	-30 (-36 to -23)	106.3 (89.5 to 126.1)	80.9 (67.8 to 95.5)	-24 (-31 to -16)	248.5 (212 to 287.9)	169.9 (147.3 to 192.5)	-32 (-38 to -23)	33.8 (22.9 to 47.8)	21.3 (14.8 to 31.3)	-37 (-47 to -24)
Alcohol use	186.1 (115.8 to 265.4)	115.7 (57.2 to 179)	-38 (-55 to -23)	*5.9 (-13.6 to 27.1)*	*-1.5 (-18.9 to 16.1)*	*-126 (-908 to 762)*	180.1 (116 to 251.7)	117.3 (62.8 to 170)	-35 (-50 to -23)	0 (0 to 0)	0 (0 to 0)	0 (0 to 0)
High fasting plasma glucose	630.5 (421.6 to 897.9)	526.7 (353.2 to 740.8)	-16 (-24 to -7)	226.5 (112.9 to 436.8)	200.7 (101.7 to 387.3)	-11 (-19 to -1)	366.8 (215.6 to 562.8)	296 (177.1 to 448.4)	-19 (-27 to -9)	37.2 (20.2 to 61.9)	30.1 (16.3 to 50.5)	-19 (-31 to -5)
High systolic blood pressure	1584.9 (1342.7 to 1820.3)	1256.9 (1071.6 to 1414.2)	-21 (-26 to -14)	488.2 (379 to 600.5)	408.3 (314.7 to 497)	-16 (-22 to -10)	983.7 (786 to 1189.8)	764.5 (631.5 to 892)	-22 (-29 to -15)	113 (79 to 160.9)	84 (59.9 to 123.7)	-26 (-35 to -14)
High body-mass index	329.1 (132.4 to 601.6)	387.5 (215.5 to 584.6)	*18 (-6 to 70)*	71.1 (27.6 to 133.9)	91.9 (49.3 to 145.4)	29 (4 to 86)	226.4 (89.9 to 413.6)	260.6 (144.3 to 399.4)	*15 (-8 to 67)*	31.6 (12.1 to 61.8)	35 (18 to 59.2)	*11 (-14 to 70)*
Dietary risks	1656.9 (1431.3 to 1901.9)	1163.4 (1004.4 to 1332)	-30 (-35 to -23)	488.3 (383.3 to 591)	372.4 (293.2 to 449.3)	-24 (-30 to -17)	1040.5 (839.2 to 1254.7)	707.1 (575 to 844.1)	-32 (-38 to -25)	128.1 (92.7 to 176.8)	83.8 (59.4 to 119.9)	-35 (-43 to -24)
Low physical activity	90.6 (30.2 to 175.3)	70.7 (24 to 135)	-22 (-28 to -15)	90.6 (30.2 to 175.3)	70.7 (24 to 135)	-22 (-28 to -15)	0 (0 to 0)	0 (0 to 0)	0 (0 to 0)	0 (0 to 0)	0 (0 to 0)	0 (0 to 0)
Impaired kidney function	248.5 (198 to 305.1)	182.6 (147.6 to 220.4)	-26 (-32 to -20)	92 (68.4 to 117.1)	71 (53.4 to 90.6)	-23 (-29 to -16)	156.5 (123.8 to 193.2)	111.6 (90.2 to 135.1)	-29 (-35 to -21)	0 (0 to 0)	0 (0 to 0)	0 (0 to 0)
High LDL cholesterol	173.8 (98.6 to 306.6)	147.7 (84.5 to 258.2)	-15 (-22 to -7)	173.8 (98.6 to 306.6)	147.7 (84.5 to 258.2)	-15 (-22 to -7)	0 (0 to 0)	0 (0 to 0)	0 (0 to 0)	0 (0 to 0)	0 (0 to 0)	0 (0 to 0)
World Bank Lower Middle Income												
All risk factors	2293.9 (2176.6 to 2406.5)	1809.2 (1721.2 to 1895)	-21 (-25 to -18)	912.3 (817 to 1020.2)	714.5 (638.7 to 801.4)	-22 (-26 to -17)	1174.1 (1047.7 to 1278.9)	931.5 (860.5 to 993.2)	-21 (-26 to -16)	207.4 (173.1 to 259.1)	163.2 (144 to 192.6)	-21 (-29 to -12)
Air pollution	431.7 (363.7 to 502)	282.1 (230.7 to 332.5)	-35 (-39 to -30)	149.3 (120.2 to 180)	98.6 (76.9 to 121.9)	-34 (-39 to -29)	237.4 (193.1 to 279.5)	154.2 (123.9 to 185.2)	-35 (-40 to -30)	45 (34.2 to 59.5)	29.3 (22.4 to 37.3)	-35 (-42 to -27)
Other environmental risks	174.8 (117.5 to 236.9)	138.6 (93.1 to 188.3)	-21 (-26 to -16)	55.7 (35.2 to 78.5)	50.7 (32.6 to 70.8)	-9 (-16 to -1)	100.9 (65.2 to 140.2)	75.2 (48.2 to 106.1)	-25 (-31 to -19)	18.3 (11 to 28.3)	12.7 (7.6 to 18.9)	-30 (-39 to -18)
Tobacco	499.4 (454.4 to 546.4)	346.6 (314.5 to 379.9)	-31 (-36 to -26)	173.5 (153.2 to 197.9)	120 (106.1 to 137.4)	-31 (-36 to -26)	275.8 (240.4 to 309.9)	192.7 (172.5 to 213.7)	-30 (-36 to -24)	50.1 (37.8 to 68.1)	33.9 (28.4 to 42.2)	-32 (-42 to -18)
Alcohol use	129.9 (84.1 to 177.3)	158 (120.2 to 200.3)	22 (0 to 62)	17.7 (-3.5 to 39.9)	31.9 (15.7 to 48.9)	*80 (-662 to 998)*	112.2 (76.6 to 150.1)	126.1 (92.2 to 162.1)	*12 (-6 to 42)*	0 (0 to 0)	0 (0 to 0)	0 (0 to 0)
High fasting plasma glucose	495.6 (336.7 to 727.4)	502.8 (350.6 to 701.9)	*1 (-7 to 11)*	216.2 (108.9 to 403.7)	212.3 (113 to 379)	*-2 (-12 to 10)*	240.2 (149.7 to 359.9)	249.8 (160 to 360.9)	*4 (-6 to 15)*	39.1 (23.8 to 63.3)	40.6 (25.3 to 61.1)	*4 (-9 to 19)*
High systolic blood pressure	1466.2 (1250.8 to 1653.1)	1199.5 (1035 to 1343.5)	-18 (-22 to -14)	556.1 (442.5 to 672.3)	447.1 (357.7 to 535.1)	-20 (-24 to -15)	772.7 (627 to 912)	639.5 (536.5 to 742.1)	-17 (-23 to -12)	137.3 (106 to 177.1)	112.9 (90.7 to 139.2)	-18 (-26 to -8)
High body-mass index	318.2 (165.8 to 518.9)	448.3 (289.2 to 620.2)	41 (16 to 81)	96.1 (51.5 to 156.5)	121.2 (75.6 to 175.8)	26 (9 to 55)	182.5 (91.7 to 304.4)	268.4 (172 to 372.8)	47 (18 to 95)	39.6 (19.9 to 66.1)	58.7 (37.4 to 83.4)	48 (18 to 101)
Dietary risks	1474.6 (1301.9 to 1649.7)	1057.7 (926.4 to 1202.3)	-28 (-32 to -25)	529 (430.5 to 630)	377.9 (309.8 to 449.5)	-29 (-32 to -24)	794.5 (655.5 to 928.5)	571.4 (477.3 to 672)	-28 (-33 to -23)	151 (120 to 192.4)	108.4 (87.5 to 132.8)	-28 (-35 to -20)
Low physical activity	104.2 (35.3 to 195.8)	79.4 (27.3 to 148)	-24 (-28 to -19)	104.2 (35.3 to 195.8)	79.4 (27.3 to 148)	-24 (-28 to -19)	0 (0 to 0)	0 (0 to 0)	0 (0 to 0)	0 (0 to 0)	0 (0 to 0)	0 (0 to 0)
Impaired kidney function	219.2 (179.7 to 263.4)	178.7 (147.7 to 214.2)	-18 (-23 to -14)	98.2 (76.4 to 122.2)	77.8 (61.3 to 96.3)	-21 (-25 to -15)	121 (97.5 to 145.9)	100.9 (82.8 to 120.7)	-17 (-22 to -11)	0 (0 to 0)	0 (0 to 0)	0 (0 to 0)
High LDL cholesterol	210.8 (116.2 to 372.3)	168.6 (97.3 to 296.7)	-20 (-24 to -14)	210.8 (116.2 to 372.3)	168.6 (97.3 to 296.7)	-20 (-24 to -14)	0 (0 to 0)	0 (0 to 0)	0 (0 to 0)	0 (0 to 0)	0 (0 to 0)	0 (0 to 0)
World Bank Upper Middle Income												
All risk factors	2632.2 (2511.5 to 2772.3)	1713.5 (1618.1 to 1812.6)	-35 (-38 to -32)	990.5 (916.9 to 1073.1)	759.4 (691.4 to 833.1)	-23 (-28 to -19)	1320.1 (1241.1 to 1474.3)	827.2 (784.3 to 866.7)	-37 (-43 to -34)	321.6 (223.9 to 367.1)	126.9 (112.9 to 137.7)	-61 (-66 to -46)
Air pollution	424 (353 to 494.8)	220.8 (177.6 to 265.2)	-48 (-52 to -45)	133 (107.3 to 159.4)	85.9 (67.6 to 105.6)	-35 (-41 to -30)	229.7 (187.8 to 275.8)	117 (94.1 to 140.3)	-49 (-55 to -45)	61.3 (40.7 to 75.5)	17.9 (13.9 to 21.8)	-71 (-75 to -59)
Other environmental risks	195.9 (132.5 to 265.1)	125.6 (83.6 to 170.2)	-36 (-41 to -31)	53.8 (33.5 to 77.1)	49.6 (31.6 to 70.2)	*-8 (-17 to 3)*	111.9 (73.7 to 156.8)	67.3 (43.7 to 94.4)	-40 (-48 to -33)	30.2 (16.3 to 44.6)	8.7 (5.3 to 12.6)	-71 (-78 to -57)
Tobacco	747.8 (691.5 to 805.9)	464.6 (431.7 to 499.7)	-38 (-41 to -34)	247.6 (225.6 to 270.8)	186.1 (169.6 to 205.7)	-25 (-31 to -19)	401.4 (363.8 to 451.8)	241.8 (224.9 to 259.2)	-40 (-45 to -35)	98.8 (69.5 to 117.6)	36.6 (31.3 to 41.1)	-63 (-69 to -50)
Alcohol use	265.1 (184.8 to 348.7)	258.5 (187.7 to 327.5)	*-2 (-23 to 29)*	*32.9 (-0.3 to 68.7)*	60.9 (30.2 to 93.4)	*85 (-200 to 712)*	232.1 (164.6 to 299.7)	197.6 (143.5 to 250.3)	*-15 (-32 to 10)*	0 (0 to 0)	0 (0 to 0)	0 (0 to 0)
High fasting plasma glucose	449.7 (313.6 to 630.1)	319.1 (221.8 to 448.8)	-29 (-34 to -24)	192 (105.4 to 346.3)	152.2 (85.5 to 260.8)	-21 (-27 to -13)	209.3 (134.9 to 295.4)	143.6 (95.9 to 199.3)	-31 (-38 to -26)	48.4 (28.7 to 71.9)	23.2 (15.5 to 32)	-52 (-59 to -35)
High systolic blood pressure	1591 (1343.6 to 1810)	1095.7 (930.5 to 1236)	-31 (-34 to -28)	582.6 (459.2 to 694.7)	464.3 (364.9 to 551.4)	-20 (-25 to -16)	810.6 (657.2 to 971.1)	546.1 (452.3 to 637.3)	-33 (-39 to -28)	197.7 (135 to 245.2)	85.3 (69.6 to 100.3)	-57 (-63 to -41)
High body-mass index	492.6 (253.8 to 789)	477.8 (295.8 to 669.6)	*-3 (-17 to 20)*	149.3 (78.4 to 236.4)	157.8 (92 to 235.1)	*6 (-6 to 22)*	272 (135.6 to 444.8)	265.5 (164.1 to 376)	*-2 (-18 to 24)*	71.3 (35.5 to 119.8)	54.5 (37 to 72.8)	*-24 (-43 to 9)*
Dietary risks	1864.7 (1684.4 to 2045.5)	1130.3 (1008.6 to 1248.1)	-39 (-43 to -36)	627.5 (536 to 718.2)	461.7 (390.1 to 530.3)	-26 (-32 to -21)	983 (850 to 1140.6)	578.7 (504.4 to 646.9)	-41 (-47 to -37)	254.2 (171.7 to 301.5)	89.9 (76.5 to 103.3)	-65 (-70 to -51)
Low physical activity	119.5 (41.7 to 223.1)	88 (30.1 to 164.7)	-26 (-31 to -21)	119.5 (41.7 to 223.1)	88 (30.1 to 164.7)	-26 (-31 to -21)	0 (0 to 0)	0 (0 to 0)	0 (0 to 0)	0 (0 to 0)	0 (0 to 0)	0 (0 to 0)
Impaired kidney function	217.5 (177.8 to 263.5)	139.3 (113.8 to 167.6)	-36 (-40 to -32)	97.8 (77 to 120.5)	68.4 (54.4 to 84.5)	-30 (-35 to -25)	119.7 (98 to 145.5)	70.9 (58.7 to 84.3)	-41 (-46 to -37)	0 (0 to 0)	0 (0 to 0)	0 (0 to 0)
High LDL cholesterol	268.1 (153.1 to 454)	203 (116 to 345.7)	-24 (-29 to -20)	268.1 (153.1 to 454)	203 (116 to 345.7)	-24 (-29 to -20)	0 (0 to 0)	0 (0 to 0)	0 (0 to 0)	0 (0 to 0)	0 (0 to 0)	0 (0 to 0)
World Bank High Income												
All risk factors	1142.6 (1081.3 to 1199.9)	555.6 (507.3 to 602.9)	-51 (-54 to -49)	552 (504.7 to 599.4)	267.9 (231 to 305.6)	-51 (-55 to -49)	443.9 (425.8 to 459)	198.4 (188 to 208.3)	-55 (-57 to -54)	146.7 (138.3 to 155.2)	89.3 (81.5 to 97.3)	-39 (-42 to -36)
Air pollution	105.1 (80.3 to 132)	43.9 (32.6 to 55.8)	-58 (-61 to -56)	43.9 (33 to 55.9)	17.9 (13.3 to 23.3)	-59 (-62 to -56)	45.2 (34.3 to 56.3)	17.8 (13.1 to 22.8)	-61 (-63 to -58)	16 (12 to 20.2)	8.3 (6 to 10.7)	-48 (-52 to -45)
Other environmental risks	37.6 (16.9 to 62.2)	14.1 (5.4 to 25.7)	-62 (-69 to -58)	16 (7.2 to 26.9)	6.6 (2.6 to 11.9)	-59 (-65 to -55)	16.7 (7.4 to 28)	5.6 (2.1 to 10.4)	-67 (-74 to -62)	4.8 (1.8 to 8.9)	1.9 (0.5 to 4)	-61 (-71 to -54)
Tobacco	388.1 (362.5 to 413.8)	129.3 (116.2 to 143.3)	-67 (-69 to -65)	161.6 (147 to 176.7)	53.7 (46 to 61.7)	-67 (-69 to -64)	163.8 (154.6 to 172.3)	49.7 (46 to 53.6)	-70 (-71 to -68)	62.8 (58.3 to 67.5)	25.9 (23.3 to 29)	-59 (-61 to -56)
Alcohol use	150.1 (101.9 to 199.9)	52.6 (27.6 to 78.7)	-65 (-76 to -57)	28.2 (-5.7 to 63.2)	5.9 (-11 to 24.1)	*-79 (-259 to 70)*	121.9 (87.1 to 153.6)	46.7 (29.8 to 62.2)	-62 (-68 to -56)	0 (0 to 0)	0 (0 to 0)	0 (0 to 0)
High fasting plasma glucose	251.7 (165.7 to 396)	152.8 (100.1 to 227.4)	-39 (-45 to -33)	135.8 (68.9 to 261.9)	81.4 (41.4 to 149.9)	-40 (-46 to -33)	91.8 (59.7 to 131.9)	52.2 (33.9 to 76.1)	-43 (-48 to -37)	24 (15.8 to 33.4)	19.2 (12.6 to 27)	-20 (-26 to -12)
High systolic blood pressure	762.2 (663.7 to 857.2)	333.7 (280 to 386.2)	-56 (-58 to -54)	352.9 (281.9 to 419.5)	153.1 (116.7 to 188.4)	-57 (-60 to -54)	306.8 (259.6 to 350.1)	124.2 (102.2 to 145.1)	-60 (-61 to -58)	102.6 (85.7 to 118.6)	56.4 (45.7 to 67.7)	-45 (-48 to -42)
High body-mass index	318.7 (198.8 to 445.6)	213.4 (150.8 to 275.8)	-33 (-40 to -21)	106.8 (62.1 to 158.7)	75.2 (49.5 to 105.3)	-30 (-38 to -16)	147.7 (91.9 to 207)	89.8 (65.4 to 114.9)	-39 (-46 to -28)	64.3 (42.4 to 86.8)	48.3 (35.7 to 61.3)	-25 (-32 to -14)
Dietary risks	608.4 (540 to 686)	303.2 (260.6 to 345.1)	-50 (-52 to -48)	263.5 (219.7 to 308.1)	132.8 (106.6 to 159.7)	-50 (-53 to -47)	249.9 (212.9 to 289.3)	112.6 (94.8 to 130.7)	-55 (-56 to -53)	95 (80.9 to 109.7)	57.8 (48.4 to 67.4)	-39 (-42 to -36)
Low physical activity	72.9 (24.9 to 137.4)	36.3 (12.1 to 68.5)	-50 (-53 to -47)	72.9 (24.9 to 137.4)	36.3 (12.1 to 68.5)	-50 (-53 to -47)	0 (0 to 0)	0 (0 to 0)	0 (0 to 0)	0 (0 to 0)	0 (0 to 0)	0 (0 to 0)
Impaired kidney function	87 (67.9 to 108.1)	41.3 (31.9 to 52.1)	-53 (-55 to -50)	49.6 (36.6 to 63.6)	24.5 (18.1 to 32)	-51 (-55 to -47)	37.4 (30.9 to 44.8)	16.8 (13.7 to 20.4)	-55 (-57 to -53)	0 (0 to 0)	0 (0 to 0)	0 (0 to 0)
High LDL cholesterol	166.3 (86.7 to 293.9)	78.8 (43.4 to 135)	-53 (-56 to -47)	166.3 (86.7 to 293.9)	78.8 (43.4 to 135)	-53 (-56 to -47)	0 (0 to 0)	0 (0 to 0)	0 (0 to 0)	0 (0 to 0)	0 (0 to 0)	0 (0 to 0)

Note: Data were extracted from [8]; All risk factors data report the aggregated values for all 11 modifiable risk factors, i.e., air pollution, other environmental risks, tobacco, alcohol use, high fasting plasma glucose, high systolic blood pressure, high body-mass index, dietary risks, low physical activity, impaired kidney function, and high LDL cholesterol; Non-significant changes are set in *italics*; Rates are defined per 100,000 people

### Hypertension

Globally, hypertension has remained the leading modifiable predictor of stroke mortality since 1990 irrespective of SES (Table 4). This is despite a 32.7% decrease in hypertension-attributed risk of age-standardised global stroke mortality rate from 1990 to 2017, which varied from 19.7% decrease in LICs to 61.7% decrease in HICs (Table 3). It has also remained the top predictor of stroke mortality in different countries being classified based on SDI (Table 4). In 2017, deaths related to ischaemic rather than haemorrhagic strokes attributable to high systolic blood pressure were more common among women than men (Additional file 3: Table S2). Overall, there is a 3.8-fold difference in rates of stroke mortality attributable to hypertension between the most-affected and the least-affected income category, from 59.1 per 100,000 in LICs to 15.4 per 100,000 in HICs.

**Table 4:**
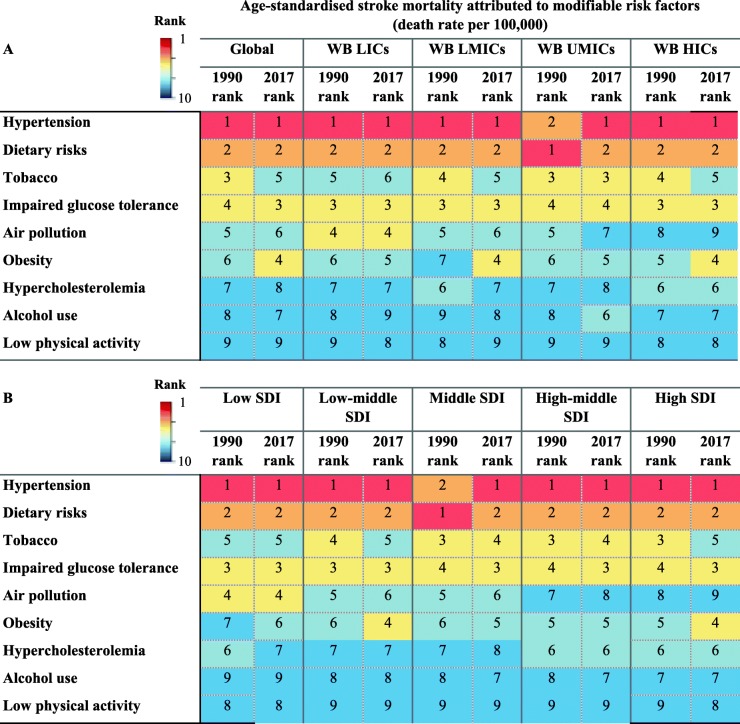
Rank of age-standardised stroke mortality rate attributable to modifiable risk factors in (A) The World Bank (WB) low-income countries (LICs), lower-middle-income countries (LMICs), upper-middle-income countries (UMICs), and high-income countries (HICs) in 1990 and 2017 and (B) in different socioeconomic status regions classified by Socio-Demographic Index (SDI) in 1990 and 2017 (extracted from [8])

Note: The order of risk factors has remained constant for an easier comparison between different socio-economical status regions

*HICs* high-income countries, *LICs* low-income countries, *LMICs* lower-middle-income countries, *UMICs* upper-middle-income countries, *SDI* Socio-Demographic Index, *WB* The World Bank

### Dietary risks

Poor dietary habits (i.e. a diet low in fibre, fruits, vegetables, legumes, whole grains, nuts and seeds, milk, calcium, or seafood, and high in red meat, eggs, processed meat, sugar-sweetened beverages, trans-fatty acids, or sodium) are globally the second leading cause of stroke mortality, irrespective of income levels or SDI (Table 4). However, from 1990 to 2017, there was a 36.7% decrease in dietary-attributed risk of age-standardised global stroke mortality rate, which varied from 28.7% decrease in LICs to 57.3% decrease in HICs (Table 3). Overall, there is a 4.3-fold difference in rates of stroke mortality attributable to dietary risks between the most-affected and the least-affected income category, from 49.2 per 100,000 in LICs to 11.4 per 100,000 in HICs.

**Fig. 2:**
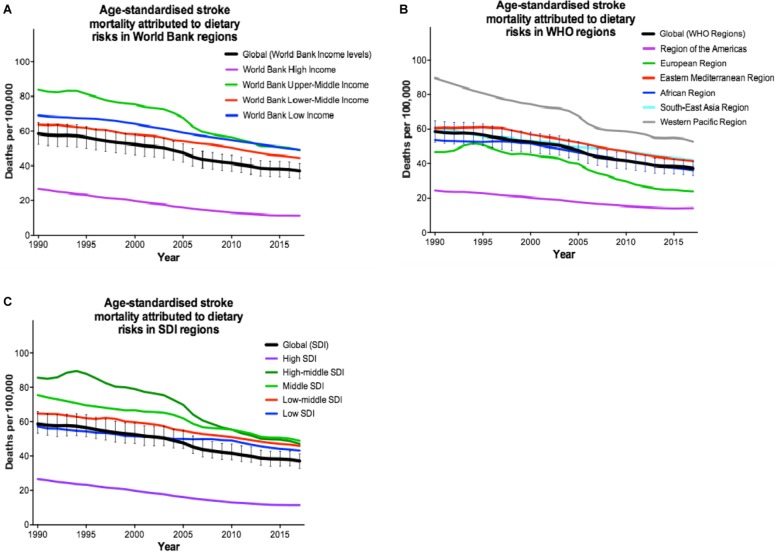
Trends in age-standardised stroke mortality rates attributable to dietary risks being classified as to **a** the World Bank income levels, **b** the World Health Organization regions (WHO), and **c** the Socio-Demographic Index (SDI) from 1990 to 2017 (extracted from [8])

In addition, dietary risks can worsen the consequences of stroke. In particular, diets low in fruits, low in whole grains, low in vegetables, high in sodium, and high in sugar-sweetened beverages increase the likelihood of global stroke mortality (Fig. 2). Worldwide, there is a downward trend in stroke events attributable to dietary risks in different SES regions (Fig. 2). The downslope became steeper in the UMICs since 2005, particularly in European and Western Pacific regions compared to the others. This suggests that better education on healthy diets is needed in these areas. It is also possible that the speed of deterioration in other cofactors may counteract the improvement in dietary risks.

### Diabetes mellitus

Diabetes and glucose intolerance-related mortality is globally the third critical risk factor of stroke mortality in 2017 (Table 4). However, the age-standardised global stroke mortality rate attributable to diabetes mellitus has decreased by 25.0% from 1990 to 2017, which varied from 4.3% decrease in LMICs to 48.2% decrease in HICs (Table 3). Overall, there is a 3.5-fold difference in rates of stroke mortality attributable to diabetes mellitus between the most-affected and the least-affected income category, from 27.1 per 100,000 in LICs to 7.8 per 100,000 in HICs.

### Obesity

Obesity is globally the fourth most influential indicator of stroke mortality, which varies slightly between different SES (Table 4). Although the age-standardised global stroke mortality rate attributable to obesity has decreased by 3.8%, although non-significantly from 1990 to 2017, more prominently in HICs (44.0%), its risk increased in both LICs and LMICs by 17.1% (statistically not significantly) and 32.7%, respectively (Table 3). This is contrary to the higher proportion of obesity observed in HICs than LMICs [13], which might be related to the occurrence of first stroke at younger ages in countries of lower-income levels (Table 5). The obesity epidemic is just more recent in middle-income countries as compared to HICs, given the ongoing epidemiological and nutritional transition happening in LMICs. Overall, there is a 2.6-fold difference in rates of stroke mortality attributable to obesity between the most-affected and the least-affected income category, from 16.1 per 100,000 in UMICs to 6.1 per 100,000 in HICs.

**Table 5:** Fractions of stroke incidence, prevalence, mortality, and DALYs lost in different age-groups and income levels in 2017

	LICs	LMICs	HMICs	HICs	Global
Incidence (%)					
< 40 years	1.4	1.4	1	1.7	*1.3*
40–64 years	10.7	11	10.9	9.8	*10.9*
≥ 65 years	87.8	87.5	88.1	88.5	*87.8*
Prevalence (%)					
< 40years	3.1	2.8	2	2.4	*2.1*
40–64 years	18.7	18.5	16	13.3	*15.2*
≥ 65 years	78.2	78.7	82	84.3	*82.7*
Mortality (%)					
< 40 years	0.8	0.5	0.3	0.2	*0.5*
40–64 years	4.4	3.9	2.7	1.5	*3.4*
≥ 65 years	94.8	95.6	97	98.3	*96*
DALYs (%)					
< 40 years	6.3	4	2.5	2.6	*4.4*
40–64 years	15.4	14.8	11.1	8.4	*13.2*
≥ 65 years	78.2	81.2	86.4	88.9	*82.4*

*DALYs* disability-adjusted life years, *HICs* high-income countries, *LICs* low-income countries, *LMICs* lower-middle-income countries, *UMICs* upper-middle-income countries

Data were extracted from [8]

### Smoking

Tobacco smoking became the fifth leading predictor of stroke in 2017 (Table 4). From 1990 to 2017, there was a 41.6% decrease in the age-standardised global stroke mortality attributable to tobacco, which varied from a 30.5% decrease in LICs to a 72.3% decrease in HICs (Table 3). However, the highest tobacco-attributed stroke mortality rate has been observed in UMICs from 1990 to 2017, which holds the third place, after hypertension and dietary risks, among all modifiable risk factors in this specific income category. Overall, there is a 4.5-fold difference in rates of stroke mortality attributable to tobacco between the most-affected and the least-affected income category, from 18.9 per 100,000 in UMICs to 4.2 per 100,000 in HICs.

### Air pollution

Air pollution is globally the sixth leading cause of stroke death with no change in its rank from 1990 to 2017 (Table 4). Its attributable risk is higher in regions with lower SES. There is a downward trend in stroke mortality attributable to air pollution in all regions of the World Bank income levels, which is steep in LICs and in UMICs. This suggests improved general awareness in these regions, in particular. However, with regard to the SDI classification, educational attainment and society population do not seem to affect the attributable risk of stroke mortality due to air pollution (Table 4). Overall, the age-standardised global stroke mortality rate attributable to air pollution has decreased by 62.3% from 1990 to 2017, which varied from 33.0% decrease in LICs to 47.4% decrease in UMICs (Table 3). Overall, there is a 1.9-fold difference in rates of stroke mortality attributable to air pollution between the most-affected and the least-affected income category, from 16.2 per 100,000 in LICs to 8.6 per 100,000 in HICs.

In 2017, ambient particulate matter pollution and household air pollution from solid fuels were globally responsible for 10.5 and 5.9 million stroke-related DALYs lost and 444.9 and 231.8 thousand stroke-related deaths, respectively (Table 6). Based on the age-standardised rates per 100,000 people worldwide, a third of the 2017 air pollution-related stroke mortality was attributable to household air pollution and two thirds of it was attributable to ambient air pollution. The portion of household air pollution dominates in LICs; it dramatically decreases in wealthier societies, in particular, UMICs and HICs, and increases in LICs and LMICs. Household air pollution is more common in females. Furthermore, in 2017, the attributable risk of haemorrhagic stroke was almost double that of ischaemic stroke worldwide.

**Table 6:** Global and regional stroke burden and mortality attributable to air pollution in 2017

	All-type stroke			Ischaemic stroke			Intracerebral haemorrhage			Subarachnoid haemorrhage		
	Number (thousand)	Age-standardised rate		Number (thousand)	Age-standardised rate		Number (thousand)	Age-standardised rate		Number (thousand)	Age-standardised rate	
	Mean (95% uncertainty interval)	Mean (95% uncertainty interval)	Female to male ratio	Mean (95% uncertainty interval)	Mean (95% uncertainty interval)	Female to male ratio	Mean (95% uncertainty interval)	Mean (95% uncertainty interval)	Female to male ratio	Mean (95% uncertainty interval)	Mean (95% uncertainty interval)	Female to male ratio
DALYs (disability-adjusted life years) per 100,000 people												
Global												
Air pollution	16,385.6 (13,224.6 to 19,514.3)	201.9 (164.1 to 240.1)	0.7	5784.9 (4581.1 to 6995.0)	72.2 (57.4 to 87.3)	0.8	8967.4 (7270.6 to 10,723.3)	109.7 (89.3 to 130.8)	0.7	1633.3 (1272.4 to 2021.0)	19.9 (15.6 to 24.6)	0.9
Ambient particulate matter pollution	10,515.5 (8248.0 to 12,877.4)	129.8 (101.6 to 159.0)	0.6	3950.2 (3040.2 to 4873.9)	49.4 (38.2 to 61.0)	0.7	5523.0 (4340.7 to 6835.3)	67.7 (53.2 to 83.2)	0.5	1042.2 (809.3 to 1314.3)	12.7 (9.9 to 16.0)	0.8
Household air pollution from solid fuels	5870.2 (4573.4 to 7263.3)	72.0 (56.0 to 89.0)	1.0	1834.6 (1390.5 to 2329.5)	22.8 (17.3 to 28.9)	1.1	3444.4 (2660.7 to 4305.5)	42.0 (32.6 to 52.6)	0.9	591.2 (443.5 to 776.6)	7.2 (5.4 to 9.4)	1.1
World Bank low income												
Air pollution	1233.7 (1026.6 to 1436.3)	372.2 (310.0 to 434.3)	1.0	346.8 (277.8 to 422.1)	117.0 (93.5 to 142.3)	1.3	785.9 (644.4 to 931.4)	229.0 (187.9 to 271.2)	0.9	100.9 (70.7 to 148.7)	26.2 (18.4 to 38.9)	0.8
Ambient particulate matter pollution	257.8 (193.9 to 334.5)	78.5 (58.5 to 102.7)	0.6	73.5 (53.8 to 97.7)	25.0 (18.0 to 33.3)	0.8	161.9 (121.9 to 210.7)	47.6 (35.9 to 63.0)	0.5	22.4 (14.5 to 33.8)	5.9 (3.8 to 9.0)	0.5
Household air pollution from solid fuels	975.9 (804.3 to 1161.0)	293.8 (241.9 to 349.1)	1.1	273.3 (215.0 to 339.1)	92.0 (71.8 to 114.4)	1.5	624.1 (505.2 to 755.4)	181.4 (145.9 to 219.7)	1.0	78.6 (53.5 to 116.6)	20.3 (13.9 to 30.6)	0.8
World Bank lower middle income												
Air pollution	6858.7 (5569.0 to 8122.8)	282.1 (230.7 to 332.5)	0.8	2210.7 (1731.5 to 2754.2)	98.6 (76.9 to 121.9)	0.9	3864.7 (3106.8 to 4631.0)	154.2 (123.9 to 185.2)	0.8	783.2 (598.3 to 991.4)	29.3 (22.4 to 37.3)	1.0
Ambient particulate matter pollution	3579.6 (2832.0 to 4445.7)	147.4 (116.4 to 182.6)	0.7	1215.2 (931.1 to 1532.2)	54.1 (41.6 to 68.1)	0.7	1961.2 (1538.8 to 2471.9)	78.2 (61.4 to 98.8)	0.6	403.2 (299.8 to 529.0)	15.1 (11.2 to 19.8)	0.8
Household air pollution from solid fuels	3279.2 (2552.6 to 4049.6)	134.7 (105.1 to 165.7)	1.1	995.6 (753.2 to 1285.3)	44.5 (34.0 to 56.9)	1.2	1903.5 (1466.3 to 2394.9)	76.0 (58.7 to 95.4)	1.0	380.1 (281.3 to 507.2)	14.2 (10.6 to 19.0)	1.2
World Bank upper middle income												
Air pollution	7302.6 (5851.4 to 8792.7)	220.8 (177.6 to 265.2)	0.6	2793.7 (2192.9 to 3426.0)	85.9 (67.6 to 105.6)	0.7	3912.0 (3133.7 to 4715.4)	117.0 (94.1 to 140.3)	0.6	596.9 (460.3 to 731.4)	17.9 (13.9 to 21.8)	0.8
Ambient particulate matter pollution	5740.9 (4467.1 to 7074.4)	173.7 (136.2 to 213.7)	0.6	2249.4 (1720.3 to 2798.2)	69.2 (53.2 to 86.0)	0.6	3021.3 (2361.5 to 3694.9)	90.4 (71.0 to 110.6)	0.5	470.2 (364.2 to 588.9)	14.1 (10.9 to 17.5)	0.7
Household air pollution from solid fuels	1561.7 (1139.5 to 2070.9)	47.1 (34.6 to 62.7)	0.9	544.3 (394.6 to 729.0)	16.7 (12.2 to 22.5)	0.9	890.7 (645.6 to 1181.3)	26.6 (19.6 to 35.4)	0.8	126.7 (91.7 to 169.8)	3.8 (2.8 to 5.0)	1.1
World Bank high income												
Air pollution	884.9 (659.1 to 1137.8)	43.9 (32.6 to 55.8)	0.7	395.6 (288.5 to 517.8)	17.9 (13.3 to 23.3)	0.7	345.0 (257.0 to 445.4)	17.8 (13.1 to 22.8)	0.6	144.3 (104.1 to 188.1)	8.3 (6.0 to 10.7)	1.1
Ambient particulate matter pollution	861.6 (640.1 to 1107.5)	42.8 (31.7 to 54.6)	0.7	383.8 (279.6 to 506.2)	17.3 (12.8 to 22.6)	0.7	336.7 (250.1 to 433.2)	17.3 (12.8 to 22.4)	0.6	141.2 (102.0 to 184.3)	8.1 (5.9 to 10.4)	1.0
Household air pollution from solid fuels	23.2 (15.3 to 33.9)	1.1 (0.8 to 1.7)	1.1	11.8 (7.6 to 17.5)	0.5 (0.3 to 0.8)	1.2	8.3 (5.5 to 12.3)	0.4 (0.3 to 0.6)	0.9	3.2 (2.1 to 4.6)	0.2 (0.1 to 0.3)	1.7
Deaths per 100,000 people												
Global												
Air pollution	676.8 (550.5 to 806.6)	8.6 (7.0 to 10.3)	0.7	264.8 (209.1 to 322.5)	3.4 (2.7 to 4.2)	0.8	358.5 (291.8 to 427.7)	4.5 (3.6 to 5.4)	0.7	53.6 (42.7 to 65.3)	0.7 (0.5 to 0.8)	0.9
Ambient particulate matter pollution	444.9 (346.0 to 549.3)	5.7 (4.4 to 7.0)	0.6	183.5 (139.9 to 228.2)	2.4 (1.8 to 3.0)	0.7	226.4 (176.0 to 279.9)	2.8 (2.2 to 3.5)	0.6	35.0 (27.3 to 44.1)	0.4 (0.3 to 0.6)	0.7
Household air pollution from solid fuels	231.8 (179.6 to 287.2)	2.9 (2.3 to 3.6)	1.0	81.2 (62.4 to 103.8)	1.0 (0.8 to 1.3)	1.1	132.1 (101.5 to 164.5)	1.6 (1.3 to 2.0)	1.0	18.5 (13.9 to 24.3)	0.2 (0.2 to 0.3)	1.1
World Bank low income												
Air pollution	45.1 (37.7 to 52.9)	16.2 (13.5 to 19.2)	1.0	14.5 (11.4 to 17.9)	5.9 (4.7 to 7.3)	1.3	27.7 (22.8 to 32.8)	9.4 (7.8 to 11.2)	0.9	2.8 (1.9 to 4.3)	0.9 (0.6 to 1.3)	0.7
Ambient particulate matter pollution	9.5 (7.1 to 12.5)	3.5 (2.5 to 4.5)	0.6	3.1 (2.2 to 4.2)	1.3 (0.9 to 1.7)	0.8	5.8 (4.4 to 7.7)	2.0 (1.5 to 2.6)	0.6	0.7 (0.4 to 1.0)	0.2 (0.1 to 0.3)	0.5
Household air pollution from solid fuels	35.5 (28.9 to 42.3)	12.8 (10.4 to 15.2)	1.1	11.4 (8.7 to 14.2)	4.7 (3.6 to 5.8)	1.5	21.9 (17.7 to 26.5)	7.4 (6.0 to 9.0)	1.0	2.2 (1.5 to 3.4)	0.7 (0.4 to 1.0)	0.8
World Bank lower middle income												
Air pollution	262.0 (215.1 to 311.6)	12.3 (10.1 to 14.7)	0.9	97.2 (76.5 to 121.1)	5.1 (4.0 to 6.3)	0.9	141.5 (113.5 to 169.5)	6.3 (5.1 to 7.5)	0.8	23.4 (18.0 to 30.1)	1.0 (0.7 to 1.2)	1.0
Ambient particulate matter pollution	136.7 (107.0 to 169.4)	6.4 (5.0 to 8.0)	0.7	53.1 (40.7 to 67.2)	2.8 (2.1 to 3.5)	0.7	71.6 (56.3 to 90.2)	3.2 (2.5 to 4.0)	0.6	12.0 (9.0 to 16.0)	0.5 (0.4 to 0.7)	0.8
Household air pollution from solid fuels	125.4 (97.5 to 155.0)	5.9 (4.6 to 7.3)	1.1	44.1 (33.9 to 56.7)	2.3 (1.8 to 2.9)	1.2	69.9 (53.5 to 87.5)	3.1 (2.4 to 3.9)	1.0	11.4 (8.5 to 15.1)	0.5 (0.3 to 0.6)	1.2
World Bank upper middle income												
Air pollution	319.5 (254.3 to 386.8)	10.2 (8.0 to 12.3)	0.7	128.9 (99.8 to 160.6)	4.2 (3.3 to 5.2)	0.7	168.7 (134.7 to 202.4)	5.3 (4.2 to 6.3)	0.6	21.9 (16.9 to 26.8)	0.7 (0.5 to 0.8)	0.8
Ambient particulate matter pollution	251.1 (193.6 to 309.2)	8.0 (6.1 to 9.8)	0.6	104.3 (79.2 to 130.1)	3.4 (2.6 to 4.3)	0.6	129.6 (100.5 to 158.7)	4.0 (3.2 to 5.0)	0.5	17.2 (13.1 to 21.4)	0.5 (0.4 to 0.7)	0.7
Household air pollution from solid fuels	68.5 (50.2 to 91.3)	2.2 (1.6 to 2.9)	0.9	24.6 (18.1 to 32.7)	0.8 (0.6 to 1.1)	0.9	39.1 (28.5 to 52.2)	1.2 (0.9 to 1.6)	0.9	4.7 (3.4 to 6.3)	0.1 (0.1 to 0.2)	1.1
World Bank high income												
Air pollution	45.6 (33.5 to 59.2)	1.9 (1.4 to 2.4)	0.7	22.5 (16.0 to 30.5)	0.9 (0.6 to 1.1)	0.7	18.0 (13.3 to 23.3)	0.8 (0.6 to 1.0)	0.6	5.1 (3.9 to 6.6)	0.3 (0.2 to 0.3)	1.0
Ambient particulate matter pollution	44.4 (32.6 to 58.0)	1.8 (1.4 to 2.4)	0.7	21.8 (15.5 to 29.6)	0.8 (0.6 to 1.1)	0.7	17.6 (12.9 to 22.8)	0.8 (0.6 to 1.0)	0.6	5.0 (3.8 to 6.5)	0.2 (0.2 to 0.3)	1.0
Household air pollution from solid fuels	1.2 (0.8 to 1.7)	0.1 (0.0 to 0.1)	1.2	0.7 (0.4 to 1.0)	0.0 (0.0 to 0.0)	1.3	0.4 (0.3 to 0.6)	0.0 (0.0 to 0.0)	1.0	0.1 (0.1 to 0.2)	0.0 (0.0 to 0.0)	1.7

Data were extracted from [8]. Rates are defined per 100,000 people

### Alcohol use

The global rank of stroke mortality related to alcohol drinking rose from the eighth rank in 1990 to the seventh in 2017 (Table 4). Although the age-standardised global stroke mortality rate attributable to alcohol use has declined by 13.5% from 1990 to 2017, particularly in HICs (70.7%), its risk increased in both LMICs (33.5%) and UMICs (3.8%) (Table 3). In 1995, the rate of alcohol use had moved up one step to the seventh rank with no change in rankings later on. With regard to sex, no change in rankings of these predictors was observed in men during the 25-year period, while in women, the age-standardised rate of alcohol use has shown a two-step decline to the seventh rank in 2017. Overall, there is a 5.3-fold difference in rates of stroke mortality attributable to alcohol use between the most-affected and the least-affected income category, from 11.1 per 100,000 in UMICs to 2.1 per 100,000 in HICs.

### Hypercholesterolemia

Hypercholesterolemia, particularly high LDL-C, became globally the eighth most important indicator of stroke mortality in 2017 (Table 4). From 1990 to 2017, the age-standardised global stroke mortality rate attributable to hypercholesterolemia decreased by 38.4%, which varied from 16.4% decrease in LICs to 64.0% decrease in HICs (Table 3). In societies of higher SES, it stands at a higher rank with regard to stroke mortality. Overall, there is a 2.8-fold difference in rates of stroke mortality attributable to hypercholesterolemia between the most-affected and the least-affected income category, from 9.2 per 100,000 in UMICs to 3.3 per 100,000 in HICs.

### Low physical activity

Low physical activity has globally remained the ninth modifiable indicator of stroke mortality since 1990, which is almost constant through the varying SES regions (Table 4). SES can be a factor in determining levels and types of physical activity, as well as facilities accessed. Nevertheless, despite the proven association of low physical activity with stroke, there is a 37.3% decline in its attributable risk of the age-standardised global stroke mortality, varying between 22.4% in LICs and 59.8% in HICs (Table 3). Overall, there is a 2.6-fold difference in rates of stroke mortality attributable to low physical activity between the most-affected and the least-affected income category, from 5.1 per 100,000 in UMICs to 2.0 per 100,000 in HICs.

### Non-modifiable predictors of stroke mortality

#### Age

**Fig. 3:**
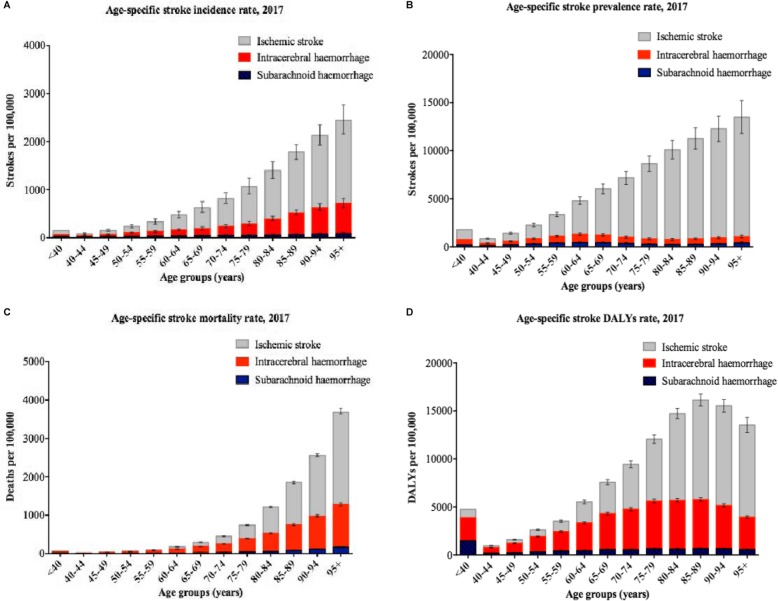
Rates of age-specific global stroke **a** incidence, **b** prevalence, **c** mortality, and **d** disability-adjusted life years (DALYs) lost in 2017 (extracted from [8])

Ageing is regarded as the most important predictor of stroke incidence and mortality, and thus, their rates increase by age (Fig. 3). Older individuals with lower SES have higher stroke incidence [14] and mortality rates [15]. Earlier, it was estimated that approximately 90% of strokes occur above age 65 years, 75% of which is > 75 years [16]. By 2017, these rates have slightly changed: 88% of global strokes occur above age 65 years, 72% of which is > 75 years (Table 5). This suggests that the age of stroke occurrence is declining and its incidence rate is increasing at younger ages. Compared to HICs, stroke prevalence at younger ages is higher in LICs (15.7% vs. 21.8% before age 65 years; 17.3% globally). In 2017, 96% of stroke-related deaths happened above age 65 years, 86% of which was > 75 years. There is also an almost twofold difference in DALYs lost at younger ages (i.e. lower than 65 years) between LICs and HICs (21.7% vs. 11.1%).

#### Sex

Risk of stroke and the risk factors differ between men and women, with almost no change in their attributed risk of stroke mortality from 1990 to 2017, particularly for tobacco use, alcohol use, obesity, and air pollution (Table 2, Additional file 3: Table S2, and Table 7). Underlying aetiology, risk factors, incidence, and outcomes of stroke vary substantially between men and women [17, 18]. Stroke occurs earlier (at 68.5 vs. 73.0 years) and more frequently in men (incidence 33% higher, and prevalence 41% higher) than in women [19], while women have more severe strokes resulting in a higher 1-month stroke fatality (25% vs. 20%) [20]. Our results suggest a pivotal role for SES in sex-stratified age-standardised stroke mortality rates (Table 8).

**Table 7:**
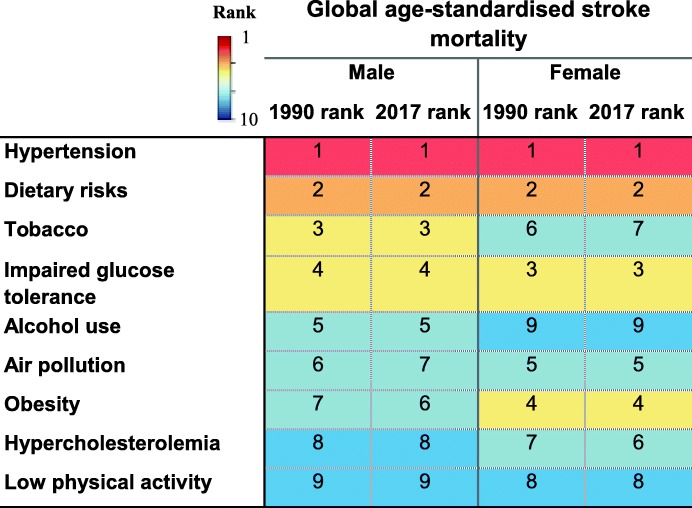
Differences in the ranks of age-standardised stroke mortality rate attributable to dietary risk factors being stratified by sex in 1990 and 2017 (extracted from [8])

**Table 8:**
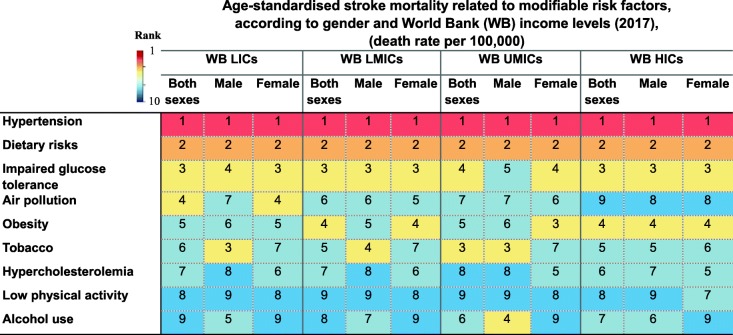
Difference in ranks of age-standardised stroke mortality rate attributable to modifiable risk factors being stratified by sex in the World Bank (WB) low-income countries (LICs), lower-middle-income countries (LMICs), upper-middle-income countries (UMICs), and high-income countries (HICs) in 1990 and 2017 (extracted from [8])

*HICs* high-income countries, *LICs* low-income countries, *LMICs* lower-middle-income countries, *UMICs* upper-middle-income countries, *WB* The World Bank

## Discussion

The results of the current study add to the body of evidence on existing disparities, gaps, and hurdles in stroke research, practice, and educational endeavours in different socioeconomic classes, countries, and regions. It appears that the age-standardised rate of stroke incidence and mortality is decreasing in all regions of varying SES (based on income-level or SDI), although more rapidly in wealthier societies. This influence is largely driven through commonly known modifiable stroke risk factors. In 2017, high systolic blood pressure and dietary risks were the top leading causes of stroke-related deaths and worldwide burden. Among the 11 modifiable risk factors, alcohol had five times higher association with haemorrhagic strokes than ischaemic strokes. Further, the rates of stroke mortality and burden were significantly higher in lower SES regions compared with HICs. Likewise, the age-standardised stroke mortality attributable to potentially modifiable risk factors is declining in almost all regions, except for obesity and alcohol use. LICs hold the worst attributable risk of stroke mortality for hypertension, dietary habits, diabetes, and air pollution, and UMICs hold the worst attributable risk of stroke mortality for obesity, tobacco use, alcohol use, hypercholesterolemia, and low physical activity. Still, HICs have almost threefold lower rates of stroke mortality attributable to modifiable risk factors compared to all other income categories.

According to the last report of the global, regional, and national burden of neurological disorders, there has been a significant reduction in the age-standardised prevalence of stroke (10% decrease) and death (30% decrease) from 1990 to 2015 [1]. By 2017, according to the present study, the age-standardised rates of global stroke prevalence and mortality exhibited a 3% increase and a 33% decrease, respectively. This reflects the evolving nature of the GBD data and the changes in stroke burden worldwide.

Based on the results of a meta-analysis of 12 population-based cohorts and case-control studies mainly in HICs, despite some limitations, reduced SES could explain more than 30% (95% confidence interval 16–48) of the stroke risk irrespective of classical vascular risk factors [21]. Based on another meta-analysis by Kerr et al. [21], blood pressure, smoking, diabetes, lipids, atrial fibrillation, history of vascular disease, obesity, and physical activity were overall associated with an additional 30–40% risk. Nevertheless, more population-based studies have been conducted since then, which improved our understanding of the predicting factors [22]. For instance, an Australian population-based study of 3077 subjects with incident stroke between 1995 and 2003 proposed that improving the SES of the most deprived inhabitants could prevent up to one fifth of strokes [23].

The disparity between stroke outcomes in LMICs and HICs can be partly explained by a combination of varying levels of general health awareness, access to healthcare, and preventative strategies starting from childhood [24, 25]. Stroke management is also challenging. Excessively increasing trends of urbanisation, pollution, smoking, obesity, less physical activity, having unhealthy diets, and ageing, particularly in LMICs, along with a much larger population with restricted access to health care may partially predict the increasing rates of stroke in these countries. This alarming increase necessitates a thorough investigation of all possible reasons, including SES determinants. Accordingly, despite many recent studies in LICs and LMICs [26–36] (Additional file 5: Table S4), we cannot confidently generalise the association of common vascular risk factors with the risk and outcome of stroke in LICs and LMICs. There used to be a ten-fold difference in rates of stroke mortality and burden between the most-affected and the least-affected countries [37]. Currently, based on our findings, this has decreased to almost threefold, which is suggestive of a substantial improvement in healthcare, preventive measures, and therapeutic outcomes. However, we should acknowledge the scantiness of population-based studies in LICs and LMICs compared to UMICs and HICs.

According to the GBD 2015 [38], there are regular shifts in stroke-related cause of death composition and population age structure with rising SDI (Table 4). Moreover, ischaemic heart disease, stroke, and diabetes are among the leading causes of premature mortality, measured as years of life lost, in most SES regions based on SDI [38]. The present study highlighted the necessity of country-specific and SES-stratified quantification of the risk of stroke attributable to each factor [22].

## Conclusions

Almost half of stroke-related mortality may be attributable to modifiable risk factors (i.e. hypertension, diabetes, dietary risks, impaired glucose intolerance, obesity, smoking, air pollution, alcohol use, hypercholesterolemia, and physical inactivity), which are mostly the outcome of poor clinical management, limited access to health care, and late detection of underlying risk factors. This necessitates allocation of resources to those modifiable risk factors with the highest impact on stroke in each SES-region. Moreover, social and economic policies to reduce inequalities in stroke care should become a health priority, particularly in less wealthy countries. These policies should focus on treating early predisposing factors and on educational programmes from childhood, which have long-lasting impacts on adulthood health. Likewise, improving worldwide primary healthcare services may have an important impact on post-stroke outcomes. It is essential to improve stroke awareness among socioeconomically deprived individuals and societies and provide equitable post-stroke medical care.

## Additional files

Additional file 1: **Text S1.** Definition of Socioeconomic status; Data gathering and search strategy; Study selection, data extraction, and analysis; Supplements to sections on various risk factors. (DOCX 40 kb)

Additional file 2: **Table S1.** Absolute numbers and rates of stroke mortality and burden attributable to modifiable risk factors. (XLSX 534 kb)

Additional file 3: **Table S2.** Sex differences in stroke-related deaths and burden attributable to modifiable risk factors. (XLSX 501 kb)

Additional file 4: **Table S3.** Age-standardised rates of stroke mortality and burden attributable to behavioural, environmental, and metabolic risks. (XLSX 501 kb)

Additional file 5: **Table S4.** Socioeconomic Status and Stroke Outcome in Low- and Middle-Income Countries. (DOCX 132 kb)

## Data Availability

The datasets generated and/or analysed during the current study are available in [8].

## Abbreviations

GBD: The Global Burden of Diseases, Injuries, and Risk Factors Study
HI: High-income
HICs: High-income countries
LICs: Low-income countries
LMICs: Lower-middle-income countries
SDI: Socio-demographic Index
SES: Socioeconomic status
UI: Uncertainty interval
UMICs: Upper-middle-income countries
WB: The World Bank

## References

[1] GBD 2015 Neurological Disorders Collaborator Group (2017). Global, regional, and national burden of neurological disorders during 1990-2015: a systematic analysis for the Global Burden of Disease Study 2015. Lancet Neurol..

[2] World Health Organization. Projections of mortality and causes of death, 2015 and 2030. Health statistics and information systems. 2013. http://www.who.int/entity/healthinfo/global_burden_disease/GHE_DthGlobal_Proj_2015_2030.xls?ua=1. Accessed 21 June 2016.

[3] World Bank Country and Lending Groups. The World Bank. 2017. https://datahelpdesk.worldbank.org/knowledgebase/articles/906519. Accessed 1 Dec 2017.

[4] Aslanyan S, Weir CJ, Lees KR, Reid JL, McInnes GT (2003). Effect of area-based deprivation on the severity, subtype, and outcome of ischemic stroke. Stroke..

[5] Wu SH, Woo J, Zhang X-H (2013). Worldwide socioeconomic status and stroke mortality: an ecological study. Int J Equity Health..

[6] Feigin VL, Forouzanfar MH, Krishnamurthi R, Mensah GA, Connor M, Bennett DA (2014). Global and regional burden of stroke during 1990-2010: findings from the Global Burden of Disease Study 2010. Lancet..

[7] Buttar HS, Li T, Ravi N (2005). Prevention of cardiovascular diseases: Role of exercise, dietary interventions, obesity and smoking cessation. Exp Clin Cardiol..

[8] Global Burden of Disease Collaborative Network. Global Burden of Disease Study 2017 (GBD 2017) Results. Seattle, United States: Institute for Health Metrics and Evaluation (IHME). 2018. http://ghdx.healthdata.org/gbd-results-tool. Accessed 19 May 2019.

[9] GBD 2017 Mortality Collaborators (2018). Global, regional, and national age-sex-specific mortality and life expectancy, 1950-2017: a systematic analysis for the Global Burden of Disease Study 2017. Lancet..

[10] GBD 2017 Disease and Injury Incidence and Prevalence Collaborators (2018). Global, regional, and national incidence, prevalence, and years lived with disability for 354 diseases and injuries for 195 countries and territories, 1990-2017: a systematic analysis for the Global Burden of Disease Study 2017. Lancet..

[11] GBD 2016 Neurology Collaborators (2019). Global, regional, and national burden of neurological disorders, 1990-2016: a systematic analysis for the Global Burden of Disease Study 2016. Lancet Neurol..

[12] GBD 2013 Mortality and Causes of Death Collaborators (2015). Global, regional, and national age-sex specific all-cause and cause-specific mortality for 240 causes of death, 1990-2013: a systematic analysis for the Global Burden of Disease Study 2013. Lancet.

[13] Clarke P, O’Malley PM, Johnston LD, Schulenberg JE (2009). Social disparities in BMI trajectories across adulthood by gender, race/ethnicity and lifetime socio-economic position: 1986-2004. Int J Epidemiol..

[14] Avendano M, Kawachi I, Van Lenthe F, Boshuizen HC, Mackenbach JP, Van den Bos GAM (2006). Socioeconomic status and stroke incidence in the US elderly: the role of risk factors in the EPESE study. Stroke..

[15] Bassuk SS, Berkman LF, Amick BC (2002). Socioeconomic status and mortality among the elderly: findings from four US communities. Am J Epidemiol..

[16] Centers for Disease Control and Prevention (CDC) (2002). State-specific mortality from stroke and distribution of place of death--United States, 1999. MMWR Morb Mortal Wkly Rep..

[17] Poorthuis MHF, Algra AM, Algra A, Kappelle LJ, Klijn CJM (2017). Female- and male-specific risk factors for stroke: a systematic review and meta-analysis. JAMA Neurol..

[18] Persky RW, Turtzo LC, McCullough LD (2010). Stroke in women: disparities and outcomes. Curr Cardiol Rep..

[19] Appelros P, Stegmayr B, Terént A (2009). Sex differences in stroke epidemiology: a systematic review. Stroke..

[20] Reeves MJ, Bushnell CD, Howard G, Gargano JW, Duncan PW, Lynch G (2008). Sex differences in stroke: epidemiology, clinical presentation, medical care, and outcomes. Lancet Neurol..

[21] Kerr GD, Slavin H, Clark D, Coupar F, Langhorne P, Stott DJ (2011). Do vascular risk factors explain the association between socioeconomic status and stroke incidence: a meta-analysis. Cerebrovasc Dis..

[22] Ferrario MM, Veronesi G, Kee F, Chambless LE, Kuulasmaa K, Jørgensen T (2017). Determinants of social inequalities in stroke incidence across Europe: a collaborative analysis of 126 635 individuals from 48 cohort studies. J Epidemiol Community Health..

[23] Heeley EL, Wei JW, Carter K, Islam MS, Thrift AG, Hankey GJ (2011). Socioeconomic disparities in stroke rates and outcome: pooled analysis of stroke incidence studies in Australia and New Zealand. Med J Aust..

[24] Liu L, Xue F, Ma J, Ma M, Long Y, Newschaffer CJ (2013). Social position and chronic conditions across the life span and risk of stroke: a life course epidemiological analysis of 22,847 American adults in ages over 50. Int J Stroke.

[25] Becher H, Palm F, Aigner A, Safer A, Urbanek C, Buggle F (2016). Socioeconomic conditions in childhood, adolescence, and adulthood and the risk of ischemic stroke. Stroke.

[26] Copstein L, Fernandes JG, Bastos GAN (2013). Prevalence and risk factors for stroke in a population of Southern Brazil. Arq Neuropsiquiatr..

[27] Engels T, Baglione Q, Audibert M, Viallefont A, Mourji F, El Alaoui Faris M (2014). Socioeconomic status and stroke prevalence in Morocco: results from the Rabat-Casablanca study. PLoS ONE..

[28] Fitzpatrick AL, Ngo QV, Ly KA, Ton TGN, Longstreth WT, Vo TT (2012). Symptoms and risk factors for stroke in a community-based observational sample in Viet Nam. J Epidemiol Glob Health..

[29] Jin H, Zhu S, Wei JW, Wang J, Liu M, Wu Y (2012). Factors associated with prehospital delays in the presentation of acute stroke in urban China. Stroke..

[30] Kamal AK, Rasheed A, Mehmood K, Murtaza M, Zaidi M, Khan M (2014). Frequency and determinants of intracranial atherosclerotic stroke in urban Pakistan. J Stroke Cerebrovasc Dis..

[31] Kumar A, Prasad M, Kathuria P, Nair P, Pandit AK, Sahu JK (2015). Low socioeconomic status is an independent risk factor for ischemic stroke: a case-control study in North Indian population. Neuroepidemiology..

[32] Menon J, Vijayakumar N, Joseph JK, David PC, Menon MN, Mukundan S (2015). Below the poverty line and non-communicable diseases in Kerala: The Epidemiology of Non-communicable Diseases in Rural Areas (ENDIRA) study. Int J Cardiol..

[33] Pandian JD, Jyotsna R, Singh R, Sylaja PN, Vijaya P, Padma MV (2011). Premorbid nutrition and short term outcome of stroke: a multicentre study from India. J Neurol Neurosurg Psychiatr..

[34] Tang X, Laskowitz DT, He L, Østbye T, Bettger JP, Cao Y (2015). Neighborhood socioeconomic status and the prevalence of stroke and coronary heart disease in rural China: a population-based study. Int J Stroke..

[35] Xu F, Ah Tse L, Yin X, Yu IT, Griffiths S (2008). Impact of socio-economic factors on stroke prevalence among urban and rural residents in Mainland China. BMC Public Health..

[36] Zhou G, Liu X, Xu G, Liu X, Zhang R, Zhu W (2006). The effect of socioeconomic status on three-year mortality after first-ever ischemic stroke in Nanjing, China. BMC Public Health..

[37] Johnston SC, Mendis S, Mathers CD (2009). Global variation in stroke burden and mortality: estimates from monitoring, surveillance, and modelling. Lancet Neurol..

[38] GBD. Global, regional, and national life expectancy, all-cause mortality, and cause-specific mortality for 249 causes of death, 1980-2015: a systematic analysis for the Global Burden of Disease Study 2015. Lancet. 2016;388:1459–544. 10.1016/S0140-6736(16)31012-1. 10.1016/S0140-6736(16)31012-1 PMC5388903 27733281

