# Supplementary material for: Socioeconomic status and stroke incidence, prevalence, mortality, and worldwide burden: an ecological analysis from the Global Burden of Disease Study 2017
Source: BMC Med. 2019 Oct 24;17:191. doi: 10.1186/s12916-019-1397-3 (PMC6813111; doi:10.1186/s12916-019-1397-3)
Supplement: Supplementary file 1 — Text S1. Definition of Socioeconomic status; Data gathering and search strategy; Study selection, data extraction, and analysis; Supplements to sections on various risk factors. (DOCX 40 kb) [file 12916_2019_1397_MOESM1_ESM.docx]

## Text S1.

### Definition of socioeconomic status

Socioeconomic status (SES) is defined as an individual’s position, economically and socially, relative to others. The term “social class” as an alternative to SES reflects many cultural and economic correlates, including occupation, income, ethnicity, geography, and other factors. The level of education, income, occupation, and quality of accommodation and properties are also regarded as the major SES determinants. Moreover, the level of access to healthcare resources can indicate SES of inhabitants of a city, country, or region. Each of these factors may have causal connections to disease occurrence and burden. Reduced SES will result in widening inequalities in resource distribution, which in turn increases the burden of chronic diseases, such as stroke. In 2016, researchers of the Global Burden of Disease (GBD) coordinated by the Institute for Health Metrics and Evaluation developed a uniform summary measure of geographical socio-demographic development, namely the Socio-Demographic Index (SDI), which is based on the combination of average income per person, educational attainment, and total fertility rate. SDI is used to compare observed patterns of health loss to the expected pattern for countries or locations with similar SDI scores, from zero to one.

### Data gathering and search strategy

We further searched PubMed/Medline as of 1 March 2018 and the references of the retrieved articles for recent population-based or hospital-based cohorts (published in English) studying the association between SES and stroke in LICs and LMICs (according to the 2017 World Bank classification of countries and regions) [[3]](http://f1000.com/work/citation?ids=6309697&pre=&suf=&sa=0). We also reported results of some population-based studies in UMICs or HICs, where applicable. We used the Medical Subject Headings (MeSH): “Socioeconomic Factors”, “Stroke”, “Adult”, “Cohort Studies”, “Risk Assessment”, “Risk Factors”, “Incidence”, “Community Health Planning”, "Home Care Services, Hospital-Based", “Smoke”, “Alcohols”, “Obesity”, "Diabetes Mellitus", "Hyperlipidemias", "Air Pollution", “Malnutrition”, and "Nutrition Surveys", in addition to their entry terms and synonyms.

###

### Study selection, data extraction, and analysis

Inclusion criteria included cross-sectional case-control analysis of population-based or hospital-based nationwide longitudinal cohorts in low- and middle-income countries based on the 2017 World Bank classification; in patients of any age with a clinical diagnosis of stroke; having reported SES determinants with any socioeconomic measures; being adjusted for both age and sex along with one or more of the stroke modifiable risk factors; having reported stroke incidence or post-stroke-care/mortality, as outcomes of interest, within a predefined period in a longitudinal setting; with no date, language, or publication restrictions. Exclusion criteria included non-original research publications; studies not fulfilling the inclusion criteria; studies with unclear adjustment of stroke risk factors and covariates; studies of cardiovascular disease with indefinite division of stroke related analysis.

## Supplements to sections on various risk factors

### Dietary risks

There are harmful dietary trends worldwide, which are accompanied by increased coronary risks and stroke, especially in younger age groups [[39]](http://f1000.com/work/citation?ids=6174296&pre=&suf=&sa=0). A healthy diet contains a variety of fruits and vegetables, whole grains, low-fat dairy products, skinless poultry and fish, nuts and legumes, non-tropical vegetable oils, and a limited intake of saturated fat, cholesterol, sodium, red meat, sweets and sugar-sweetened beverages [[40]](http://f1000.com/work/citation?ids=5991939&pre=&suf=&sa=0). Sugar, red meat, and eggs are to be blamed the most for an unhealthy diet, which dramatically increase the risk of vascular events [[39]](http://f1000.com/work/citation?ids=6174296&pre=&suf=&sa=0). For instance, in the United States, among diabetics, consuming an egg a day increased vascular events by only two-fold [[41–43]](http://f1000.com/work/citation?ids=5354347,6174332,6174333&pre=&pre=&pre=&suf=&suf=&suf=&sa=0,0,0), on a background of a very unhealthy diet: in the US only 0.1% of persons consume a healthy diet, and only 8.3% consume a somewhat healthy diet [[44]](http://f1000.com/work/citation?ids=1153598&pre=&suf=&sa=0). In Greece, however, where the Mediterranean diet is the norm, the harm from eggs was more evident: among Greek diabetics, an egg a day increased coronary risk 5-fold, and even 10 grams a day of egg (a sixth of a large egg) increased coronary risk by 54% [[41–43]](http://f1000.com/work/citation?ids=5354347,6174332,6174333&pre=&pre=&pre=&suf=&suf=&suf=&sa=0,0,0). In China, with increased prosperity in the past 15 years, an increase in consumption of meat and eggs, and a decline in consumption of fruits and vegetables, were associated with a 26.6% increase in stroke mortality and a 213% increase in coronary mortality between 2003 and 2013 [[45]](http://f1000.com/work/citation?ids=6265339&pre=&suf=&sa=0).

Based on a recent Cochrane systematic review, there is some uncertainty regarding the effects of a Mediterranean diet on cardiovascular risk factors for both primary and secondary prevention [[46]](http://f1000.com/work/citation?ids=7095496&pre=&suf=&sa=0). Evidence from the Primary Prevention of Cardiovascular Disease with a Mediterranean Diet (PREDIMED) study indicated that a Mediterranean diet supplemented with mixed nuts reduced stroke by 46% in 5 years, while the Mediterranean diet supplemented with extra-virgin olive oil reduced stroke by 35%, compared to a diet aiming for low fat intake, in high-risk primary prevention patients [[47]](http://f1000.com/work/citation?ids=5413718&pre=&suf=&sa=0). Although most of the studies on this topic do not fully comply with contemporary methodologic quality standards [[48]](http://f1000.com/work/citation?ids=6209111&pre=&suf=&sa=0), the strongest evidence for dietary prevention of stroke and myocardial infarction is with the Mediterranean diet, a mostly vegetarian diet high in olive oil, whole grains, fruits, vegetables and legumes, and low in meat and dairy products [[39, 49–51]](http://f1000.com/work/citation?ids=6174296,5736653,6209107,4097381&pre=&pre=&pre=&pre=&suf=&suf=&suf=&suf=&sa=0,0,0,0).

### Diabetes mellitus

A longitudinal study observed that a low SES during childhood is associated with an increased risk of diabetes, independent of adulthood SES [[52]](http://f1000.com/work/citation?ids=2478966&pre=&suf=&sa=0). Two population-based studies from Ontario, Canada [[53, 54]](http://f1000.com/work/citation?ids=2478979,2479005&pre=&pre=&suf=&suf=&sa=0,0) verified a widening gap in SES-related stroke mortality in diabetic patients between higher- and lower-income populations. In a multicenter population-based cohort of Latinos (n=15,079), cardiovascular risk factors, including hyperlipidemia, hypertension, obesity, hyperglycemia/diabetes, and cigarette smoking were significantly more prevalent in participants with low-SES (i.e. with low education or income) [[55]](http://f1000.com/work/citation?ids=1377171&pre=&suf=&sa=0). Among those, diabetes mellitus has shown the strongest association with stroke in both sexes after adjustment for age, sex, and some other risk factors [[55]](http://f1000.com/work/citation?ids=1377171&pre=&suf=&sa=0). In a study of almost 25,000 Swedish persons with type 1 diabetes, low SES based on income, education, marital status, and comorbidities was associated with a 2–3 times higher risk of stroke and death [[56]](http://f1000.com/work/citation?ids=2539242&pre=&suf=&sa=0). Likewise, in a nationally representative cohort study in the United States (n=527,426) [[57]](http://f1000.com/work/citation?ids=1156774&pre=&suf=&sa=0) lower levels of education and income were also associated with type 2 diabetes-related mortality. This is partly because individuals with a low SES are at a higher risk for developing diabetes, remaining undiagnosed, and subsequently untreated or poorly controlled. Therefore, the increasing cost of medications in diabetes highlights the necessity of prevention policies; especially screening programs, along with improving the public drug coverage, to lessen inequalities between target population with low and high SES.

### Obesity

Obesity is associated with socioeconomic inequality in LMICs [[58]](http://f1000.com/work/citation?ids=548845&pre=&suf=&sa=0), and is an established risk factor for stroke incidence [[59]](http://f1000.com/work/citation?ids=2478961&pre=&suf=&sa=0) and mortality [[60]](http://f1000.com/work/citation?ids=1629689&pre=&suf=&sa=0). Each 5 kg/m^2^ increase in BMI, within the range of 25–50 kg/m^2^ is associated with about 40% higher stroke mortality [[60]](http://f1000.com/work/citation?ids=1629689&pre=&suf=&sa=0). Stroke-related deaths attributable to obesity are higher in HICs (Figure 3,A), despite the fact that fewer patients may not receive lipid-lowering medications in HICs [[61–63]](http://f1000.com/work/citation?ids=2478916,2478955,2478968&pre=&pre=&pre=&suf=&suf=&suf=&sa=0,0,0) Thus, with an alarming increase in the rate of obesity, particularly in wealthier countries, it is necessary to take measures in controlling its growth. We should also consider that stroke mortality increases in those with very low BMIs. Compared to a high birth weight (>4kg), a low birth weight (<2.5 kg) is associated with a 2-fold higher stroke risk [[64]](http://f1000.com/work/citation?ids=2478937&pre=&suf=&sa=0), which necessitates paying attention to both malnutrition and obesity.

### Smoking

Available evidence shows strong associations between SES, smoking, and stroke in adulthood [[65, 66]](http://f1000.com/work/citation?ids=1987231,2478900&pre=&pre=&suf=&suf=&sa=0,0). The arterial harm from smoking is probably due to carbon monoxide and cyanide. It is likely that smoking of marijuana may have similar effects [[67]](http://f1000.com/work/citation?ids=6282005&pre=&suf=&sa=0). According to social cognitive theory, children with low SES tend to follow family and peers in initiating smoking [[68]](http://f1000.com/work/citation?ids=2478905&pre=&suf=&sa=0). The risk of stroke with smoking was is probably underestimated in studies that do not take account of passive (second-hand) smoke, which is difficult to assess. In a study, which did so, Bonita et al [[69]](http://f1000.com/work/citation?ids=5886461&pre=&suf=&sa=0) reported that active smoking was associated with a 6-fold increase in stroke risk, whereas daily second-hand smoke (at home or at work) increased stroke 1.8-fold. Two longitudinal studies [[70, 71]](http://f1000.com/work/citation?ids=2478912,2478947&pre=&pre=&suf=&suf=&sa=0,0) on socioeconomically deprived families suggest that childhood physical and psychosocial environment predisposes tendency to smoking in adulthood. Overall, prevention of smoking and tobacco use may significantly decrease stroke risk.

### Air pollution

Available evidence indicates strong associations between both short-term and long-term exposure to air pollution and mortality and morbidity after stroke [[72–74]](http://f1000.com/work/citation?ids=1147034,2478901,2478903&pre=&pre=&pre=&suf=&suf=&suf=&sa=0,0,0). In particular, almost a third of global stroke burden is attributable to air pollution (34% in LMICs vs. 10% in HICs) [[75]](http://f1000.com/work/citation?ids=1592788&pre=&suf=&sa=0). Long-term exposure to air pollution may result in chronic systemic oxidative stress and inflammation, and thus endothelial dysfunction, atherosclerosis, and cerebrovascular thrombosis [[76, 77]](http://f1000.com/work/citation?ids=2478985,2479000&pre=&pre=&suf=&suf=&sa=0,0). Community SES is inversely associated with air pollution, and the relation is area-based and sometimes pollutant-specific [[78]](http://f1000.com/work/citation?ids=2478904&pre=&suf=&sa=0). This may be partially explained by the following: globalization would drive industrial and polluted areas to poorer properties; those with lower social class are more likely to live in more polluted areas; and since they may have less access to medical care and preventive materials, they are more susceptible to the adverse consequences of air pollution [[78]](http://f1000.com/work/citation?ids=2478904&pre=&suf=&sa=0).

### Alcohol

Data from a large 43-year longitudinal study of 11,644 members of the population-based Swedish Twin Registry showed that compared to very-light drinkers (<0.5 drinks/day), heavy drinkers (>2 drinks/day) at midlife are at a higher risk of stroke (prevalence 29%; hazard ratio 1.34), even more than hypertension and diabetes [[79]](http://f1000.com/work/citation?ids=2478931&pre=&suf=&sa=0). Moreover, compared with non-drinkers, stroke occurred almost five years earlier in heavy drinkers; irrespective of genetic or other lifestyle factors [[79]](http://f1000.com/work/citation?ids=2478931&pre=&suf=&sa=0). Results of a 13-year prospective study of 2,336 Japanese men, free from cardiovascular disease and alcohol drinking, showed that alcohol is linearly associated with hypertension, and thus cardiovascular disease risk also linearly increased [[80]](http://f1000.com/work/citation?ids=2478995&pre=&suf=&sa=0). Likewise, in a population-based study of 2,609 white Americans, drinking on a daily basis or mostly without food almost doubled the risk of hypertension, compared with lifetime abstainers [[81]](http://f1000.com/work/citation?ids=6209235&pre=&suf=&sa=0). Although the pattern of alcohol drinking is independent of SES [[82]](http://f1000.com/work/citation?ids=2478974&pre=&suf=&sa=0), low-SES may potentiate vulnerable cases to alcohol abuse [[83, 84]](http://f1000.com/work/citation?ids=2478956,2478964&pre=&pre=&suf=&suf=&sa=0,0). The association between alcohol and stroke is likely to be dose-dependent and age-dependent and should be discussed in the context of ethnicity differences [[85]](http://f1000.com/work/citation?ids=6764758&pre=&suf=&sa=0).

### Hyperlipidemia

Hyperlipidemia is common in overweight/obese individuals, and it is associated with increased stroke risk [[86, 87]](http://f1000.com/work/citation?ids=2478913,2478928&pre=&pre=&suf=&suf=&sa=0,0). Hyperlipidemia is more prevalent in, but not limited to, high SES [[88]](http://f1000.com/work/citation?ids=2378942&pre=&suf=&sa=0). By the improvement of SES in developing countries, the burden of obesity tends to shift towards those with lower SES [[88]](http://f1000.com/work/citation?ids=2378942&pre=&suf=&sa=0). In LMICs, more than half of patients with stroke and related risk factors are not adequately treated for hyperlipidemia [[89, 90]](http://f1000.com/work/citation?ids=2478910,2478929&pre=&pre=&suf=&suf=&sa=0,0). Thus, the consequences of hyperlipidemia are expected to be worse in LMICs than in HICs.

### Low physical activity

Physical inactivity is a major risk factor for stroke; it contributes to most other risk factors, including diabetes, obesity, high blood pressure, and high blood cholesterol [[7]](http://f1000.com/work/citation?ids=3727020&pre=&suf=&sa=0). Given the high prevalence of physical inactivity and its importance as a risk factor for cardiovascular disease and other conditions, reducing rates of physical inactivity has enormous potential to improve health. Contrarily, physical activity has primary and secondary prevention effects against the risk of stroke and its mortality and morbidity, particularly when it lasts longer than four months [[91–93]](http://f1000.com/work/citation?ids=6293961,6293963,6293964&pre=&pre=&pre=&suf=&suf=&suf=&sa=0,0,0). A 10-year cohort study in over 16,000 healthy men demonstrated that those in the high-fitness groups experienced a 68% lower risk of stroke and death than those in the lowest-fitness group [[94]](http://f1000.com/work/citation?ids=6293962&pre=&suf=&sa=0).

### References S1.

[3. World Bank Country and Lending Groups. The World Bank. 2017. https://datahelpdesk.worldbank.org/knowledgebase/articles/906519. Accessed 1 Dec 2017.](http://f1000.com/work/bibliography/6309697)

[7. Buttar HS, Li T, Ravi N. Prevention of cardiovascular diseases: Role of exercise, dietary interventions, obesity and smoking cessation. Exp Clin Cardiol. 2005;10:229–49.](http://f1000.com/work/bibliography/3727020)

[39. Spence JD. Diet for stroke prevention. Stroke Vasc Neurol. 2018;3:44–50. doi:10.1136/svn-2017-000130.](http://f1000.com/work/bibliography/6174296)

[40. de Oliveira Otto MC, Anderson CAM, Dearborn JL, Ferranti EP, Mozaffarian D, Rao G, et al. Dietary diversity: implications for obesity prevention in adult populations: A science advisory from the american heart association. Circulation. 2018;138:e160–8. doi:10.1161/CIR.0000000000000595.](http://f1000.com/work/bibliography/5991939)

[41. Hu FB, Stampfer MJ, Rimm EB, Manson JE, Ascherio A, Colditz GA, et al. A prospective study of egg consumption and risk of cardiovascular disease in men and women. JAMA. 1999;281:1387–94.](http://f1000.com/work/bibliography/5354347)

[42. Qureshi AI, Suri FK, Ahmed S, Nasar A, Divani AA, Kirmani JF. Regular egg consumption does not increase the risk of stroke and cardiovascular diseases. Med Sci Monit. 2007;13:CR1-8.](http://f1000.com/work/bibliography/6174332)

[43. Trichopoulou A, Psaltopoulou T, Orfanos P, Trichopoulos D. Diet and physical activity in relation to overall mortality amongst adult diabetics in a general population cohort. J Intern Med. 2006;259:583–91. doi:10.1111/j.1365-2796.2006.01638.x.](http://f1000.com/work/bibliography/6174333)

[44. Mozaffarian D, Benjamin EJ, Go AS, Arnett DK, Blaha MJ, Cushman M, et al. Heart Disease and Stroke Statistics-2016 Update: A Report From the American Heart Association. Circulation. 2016;133:e38-360. doi:10.1161/CIR.0000000000000350.](http://f1000.com/work/bibliography/1153598)

[45. Weiwei C, Runlin G, Lisheng L, Manlu Z, Wen W, Yongjun W, et al. Outline of the report on cardiovascular diseases in China, 2014. Eur Heart J Suppl. 2016;18 Suppl F:F2–11. doi:10.1093/eurheartj/suw030.](http://f1000.com/work/bibliography/6265339)

[46. Rees K, Takeda A, Martin N, Ellis L, Wijesekara D, Vepa A, et al. Mediterranean-style diet for the primary and secondary prevention of cardiovascular disease. Cochrane Database Syst Rev. 2019;3:CD009825. doi:10.1002/14651858.CD009825.pub3.](http://f1000.com/work/bibliography/7095496)

[47. Estruch R, Ros E, Salas-Salvadó J, Covas M-I, Corella D, Arós F, et al. Primary Prevention of Cardiovascular Disease with a Mediterranean Diet Supplemented with Extra-Virgin Olive Oil or Nuts. N Engl J Med. 2018;378:e34. doi:10.1056/NEJMoa1800389.](http://f1000.com/work/bibliography/5413718)

[48. Huedo-Medina TB, Garcia M, Bihuniak JD, Kenny A, Kerstetter J. Methodologic quality of meta-analyses and systematic reviews on the Mediterranean diet and cardiovascular disease outcomes: a review. Am J Clin Nutr. 2016;103:841–50. doi:10.3945/ajcn.115.112771.](http://f1000.com/work/bibliography/6209111)

[49. Rees K, Hartley L, Flowers N, Clarke A, Hooper L, Thorogood M, et al. “Mediterranean” dietary pattern for the primary prevention of cardiovascular disease. Cochrane Database Syst Rev. 2013;:CD009825. doi:10.1002/14651858.CD009825.pub2.](http://f1000.com/work/bibliography/5736653)

[50. Bonaccio M, Di Castelnuovo A, Costanzo S, Gialluisi A, Persichillo M, Cerletti C, et al. Mediterranean diet and mortality in the elderly: a prospective cohort study and a meta-analysis. Br J Nutr. 2018;120:841–54. doi:10.1017/S0007114518002179.](http://f1000.com/work/bibliography/6209107)

[51. Nordmann AJ, Suter-Zimmermann K, Bucher HC, Shai I, Tuttle KR, Estruch R, et al. Meta-analysis comparing Mediterranean to low-fat diets for modification of cardiovascular risk factors. Am J Med. 2011;124:841-51.e2. doi:10.1016/j.amjmed.2011.04.024.](http://f1000.com/work/bibliography/4097381)

[52. Tamayo T, Christian H, Rathmann W. Impact of early psychosocial factors (childhood socioeconomic factors and adversities) on future risk of type 2 diabetes, metabolic disturbances and obesity: a systematic review. BMC Public Health. 2010;10:525. doi:10.1186/1471-2458-10-525.](http://f1000.com/work/bibliography/2478966)

[53. Booth GL, Bishara P, Lipscombe LL, Shah BR, Feig DS, Bhattacharyya O, et al. Universal drug coverage and socioeconomic disparities in major diabetes outcomes. Diabetes Care. 2012;35:2257–64. doi:10.2337/dc12-0364.](http://f1000.com/work/bibliography/2478979)

[54. Lipscombe LL, Austin PC, Manuel DG, Shah BR, Hux JE, Booth GL. Income-related differences in mortality among people with diabetes mellitus. CMAJ. 2010;182:E1–17. doi:10.1503/cmaj.090495.](http://f1000.com/work/bibliography/2479005)

[55. Daviglus ML, Talavera GA, Avilés-Santa ML, Allison M, Cai J, Criqui MH, et al. Prevalence of major cardiovascular risk factors and cardiovascular diseases among Hispanic/Latino individuals of diverse backgrounds in the United States. JAMA. 2012;308:1775–84. doi:10.1001/jama.2012.14517.](http://f1000.com/work/bibliography/1377171)

[56. Rawshani A, Svensson A-M, Rosengren A, Eliasson B, Gudbjörnsdottir S. Impact of socioeconomic status on cardiovascular disease and mortality in 24,947 individuals with type 1 diabetes. Diabetes Care. 2015;38:1518–27. doi:10.2337/dc15-0145.](http://f1000.com/work/bibliography/2539242)

[57. Saydah S, Lochner K. Socioeconomic status and risk of diabetes-related mortality in the U.S. Public Health Rep. 2010;125:377–88.](http://f1000.com/work/bibliography/1156774)

[58. Dinsa GD, Goryakin Y, Fumagalli E, Suhrcke M. Obesity and socioeconomic status in developing countries: a systematic review. Obes Rev. 2012;13:1067–79. doi:10.1111/j.1467-789X.2012.01017.x.](http://f1000.com/work/bibliography/548845)

[59. Song Y-M, Sung J, Davey Smith G, Ebrahim S. Body mass index and ischemic and hemorrhagic stroke: a prospective study in Korean men. Stroke. 2004;35:831–6. doi:10.1161/01.STR.0000119386.22691.1C.](http://f1000.com/work/bibliography/2478961)

[60. Prospective Studies Collaboration, Whitlock G, Lewington S, Sherliker P, Clarke R, Emberson J, et al. Body-mass index and cause-specific mortality in 900 000 adults: collaborative analyses of 57 prospective studies. Lancet. 2009;373:1083–96. doi:10.1016/S0140-6736(09)60318-4.](http://f1000.com/work/bibliography/1629689)

[61. Primatesta P, Poulter NR. Levels of dyslipidaemia and improvement in its management in England: results from the Health Survey for England 2003. Clin Endocrinol (Oxf). 2006;64:292–8. doi:10.1111/j.1365-2265.2006.02459.x.](http://f1000.com/work/bibliography/2478916)

[62. Sheng X, Murphy MJ, MacDonald TM, Wei L. Effect of statins on total cholesterol concentrations and cardiovascular outcomes in patients with diabetes mellitus: a population-based cohort study. Eur J Clin Pharmacol. 2012;68:1201–8. doi:10.1007/s00228-012-1234-5.](http://f1000.com/work/bibliography/2478955)

[63. Kotseva K, Wood D, De Backer G, De Bacquer D, Pyörälä K, Keil U, et al. EUROASPIRE III: a survey on the lifestyle, risk factors and use of cardioprotective drug therapies in coronary patients from 22 European countries. Eur J Cardiovasc Prev Rehabil. 2009;16:121–37. doi:10.1097/HJR.0b013e3283294b1d.](http://f1000.com/work/bibliography/2478968)

[64. Goldstein LB, Adams R, Alberts MJ, Appel LJ, Brass LM, Bushnell CD, et al. Primary prevention of ischemic stroke: a guideline from the American Heart Association/American Stroke Association Stroke Council: cosponsored by the Atherosclerotic Peripheral Vascular Disease Interdisciplinary Working Group; Cardiovascular Nursing Council; Clinical Cardiology Council; Nutrition, Physical Activity, and Metabolism Council; and the Quality of Care and Outcomes Research Interdisciplinary Working Group. Circulation. 2006;113:e873-923. doi:10.1161/01.STR.0000223048.70103.F1.](http://f1000.com/work/bibliography/2478937)

[65. Nordahl H, Osler M, Frederiksen BL, Andersen I, Prescott E, Overvad K, et al. Combined effects of socioeconomic position, smoking, and hypertension on risk of ischemic and hemorrhagic stroke. Stroke. 2014;45:2582–7. doi:10.1161/STROKEAHA.114.005252.](http://f1000.com/work/bibliography/1987231)

[66. Kuper H, Adami H-O, Theorell T, Weiderpass E. The socioeconomic gradient in the incidence of stroke: a prospective study in middle-aged women in Sweden. Stroke. 2007;38:27–33. doi:10.1161/01.STR.0000251805.47370.91.](http://f1000.com/work/bibliography/2478900)

[67. Hackam DG. Cannabis and stroke: systematic appraisal of case reports. Stroke. 2015;46:852–6. doi:10.1161/STROKEAHA.115.008680.](http://f1000.com/work/bibliography/6282005)

[68. Bandura A. Social foundations of thought and action: A social cognitive theory. Englewood Cliffs, N.J: Prentice-Hall.](http://f1000.com/work/bibliography/2478905)

[69. Bonita R, Duncan J, Truelsen T, Jackson RT, Beaglehole R. Passive smoking as well as active smoking increases the risk of acute stroke. Tob Control. 1999;8:156–60.](http://f1000.com/work/bibliography/5886461)

[70. Fergusson DM, Boden JM, Horwood LJ. The developmental antecedents of illicit drug use: evidence from a 25-year longitudinal study. Drug Alcohol Depend. 2008;96:165–77. doi:10.1016/j.drugalcdep.2008.03.003.](http://f1000.com/work/bibliography/2478912)

[71. Poonawalla IB, Kendzor DE, Owen MT, Caughy MO. Family income trajectory during childhood is associated with adolescent cigarette smoking and alcohol use. Addict Behav. 2014;39:1383–8. doi:10.1016/j.addbeh.2014.05.005.](http://f1000.com/work/bibliography/2478947)

[72. Shah ASV, Lee KK, McAllister DA, Hunter A, Nair H, Whiteley W, et al. Short term exposure to air pollution and stroke: systematic review and meta-analysis. BMJ. 2015;350:h1295. doi:10.1136/bmj.h1295.](http://f1000.com/work/bibliography/1147034)

[73. Donaldson K, Duffin R, Langrish JP, Miller MR, Mills NL, Poland CA, et al. Nanoparticles and the cardiovascular system: a critical review. Nanomedicine (Lond). 2013;8:403–23. doi:10.2217/nnm.13.16.](http://f1000.com/work/bibliography/2478901)

[74. Scheers H, Jacobs L, Casas L, Nemery B, Nawrot TS. Long-Term Exposure to Particulate Matter Air Pollution Is a Risk Factor for Stroke: Meta-Analytical Evidence. Stroke. 2015;46:3058–66. doi:10.1161/STROKEAHA.115.009913.](http://f1000.com/work/bibliography/2478903)

[75. Feigin VL, Roth GA, Naghavi M, Parmar P, Krishnamurthi R, Chugh S, et al. Global burden of stroke and risk factors in 188 countries, during 1990-2013: a systematic analysis for the Global Burden of Disease Study 2013. Lancet Neurol. 2016;15:913–24. doi:10.1016/S1474-4422(16)30073-4.](http://f1000.com/work/bibliography/1592788)

[76. Nemmar A, Hoylaerts MF, Hoet PHM, Nemery B. Possible mechanisms of the cardiovascular effects of inhaled particles: systemic translocation and prothrombotic effects. Toxicol Lett. 2004;149:243–53. doi:10.1016/j.toxlet.2003.12.061.](http://f1000.com/work/bibliography/2478985)

[77. Carnethon M, Whitsel LP, Franklin BA, Kris-Etherton P, Milani R, Pratt CA, et al. Worksite wellness programs for cardiovascular disease prevention: a policy statement from the American Heart Association. Circulation. 2009;120:1725–41. doi:10.1161/CIRCULATIONAHA.109.192653.](http://f1000.com/work/bibliography/2479000)

[78. Hajat A, Hsia C, O’Neill MS. Socioeconomic disparities and air pollution exposure: a global review. Curr Environ Health Rep. 2015;2:440–50. doi:10.1007/s40572-015-0069-5.](http://f1000.com/work/bibliography/2478904)

[79. Kadlecová P, Andel R, Mikulík R, Handing EP, Pedersen NL. Alcohol consumption at midlife and risk of stroke during 43 years of follow-up: cohort and twin analyses. Stroke. 2015;46:627–33. doi:10.1161/STROKEAHA.114.006724.](http://f1000.com/work/bibliography/2478931)

[80. Higashiyama A, Okamura T, Watanabe M, Kokubo Y, Wakabayashi I, Okayama A, et al. Alcohol consumption and cardiovascular disease incidence in men with and without hypertension: the Suita study. Hypertens Res. 2013;36:58–64. doi:10.1038/hr.2012.133.](http://f1000.com/work/bibliography/2478995)

[81. Stranges S, Wu T, Dorn JM, Freudenheim JL, Muti P, Farinaro E, et al. Relationship of alcohol drinking pattern to risk of hypertension: a population-based study. Hypertension. 2004;44:813–9. doi:10.1161/01.HYP.0000146537.03103.f2.](http://f1000.com/work/bibliography/6209235)

[82. Huckle T, You RQ, Casswell S. Socio-economic status predicts drinking patterns but not alcohol-related consequences independently. Addiction. 2010;105:1192–202. doi:10.1111/j.1360-0443.2010.02931.x.](http://f1000.com/work/bibliography/2478974)

[83. Fone D, Greene G, Farewell D, White J, Kelly M, Dunstan F. Common mental disorders, neighbourhood income inequality and income deprivation: small-area multilevel analysis. Br J Psychiatry. 2013;202:286–93. doi:10.1192/bjp.bp.112.116178.](http://f1000.com/work/bibliography/2478956)

[84. Hamdi NR, Krueger RF, South SC. Socioeconomic status moderates genetic and environmental effects on the amount of alcohol use. Alcohol Clin Exp Res. 2015;39:603–10. doi:10.1111/acer.12673.](http://f1000.com/work/bibliography/2478964)

[85. Millwood IY, Walters RG, Mei XW, Guo Y, Yang L, Bian Z, et al. Conventional and genetic evidence on alcohol and vascular disease aetiology: a prospective study of 500 000 men and women in China. Lancet. 2019;393:1831–42. doi:10.1016/S0140-6736(18)31772-0.](http://f1000.com/work/bibliography/6764758)

[86. Sacco RL, Liao JK. Drug Insight: statins and stroke. Nat Clin Pract Cardiovasc Med. 2005;2:576–84. doi:10.1038/ncpcardio0348.](http://f1000.com/work/bibliography/2478913)

[87. Subramanian S, Chait A. Hypertriglyceridemia secondary to obesity and diabetes. Biochim Biophys Acta. 2012;1821:819–25. doi:10.1016/j.bbalip.2011.10.003.](http://f1000.com/work/bibliography/2478928)

[88. Monteiro CA, Moura EC, Conde WL, Popkin BM. Socioeconomic status and obesity in adult populations of developing countries: a review. Bull World Health Organ. 2004;82:940–6. doi:/S0042-96862004001200011.](http://f1000.com/work/bibliography/2378942)

[89. Hu Z, Zaman MJ, Wang J, Peacock JL, Chen R. Correlates of Untreated Hypercholesterolemia in Older Adults: A Community-Based Household Survey in China. PLoS ONE. 2015;10:e0131318. doi:10.1371/journal.pone.0131318.](http://f1000.com/work/bibliography/2478910)

[90. Wu J, Zhu S, Yao GL, Mohammed MA, Marshall T. Patient factors influencing the prescribing of lipid lowering drugs for primary prevention of cardiovascular disease in UK general practice: a national retrospective cohort study. PLoS ONE. 2013;8:e67611. doi:10.1371/journal.pone.0067611.](http://f1000.com/work/bibliography/2478929)

[91. Prior PL, Suskin N. Exercise for stroke prevention. Stroke Vasc Neurol. 2018;3:59–68. doi:10.1136/svn-2018-000155.](http://f1000.com/work/bibliography/6293961)

[92. D’Isabella NT, Shkredova DA, Richardson JA, Tang A. Effects of exercise on cardiovascular risk factors following stroke or transient ischemic attack: a systematic review and meta-analysis. Clin Rehabil. 2017;31:1561–72. doi:10.1177/0269215517709051.](http://f1000.com/work/bibliography/6293963)

[93. Deijle IA, Van Schaik SM, Van Wegen EEH, Weinstein HC, Kwakkel G, Van den Berg-Vos RM. Lifestyle Interventions to Prevent Cardiovascular Events After Stroke and Transient Ischemic Attack: Systematic Review and Meta-Analysis. Stroke. 2017;48:174–9. doi:10.1161/STROKEAHA.116.013794.](http://f1000.com/work/bibliography/6293964)

[94. Lee CD, Blair SN. Cardiorespiratory fitness and stroke mortality in men. Med Sci Sports Exerc. 2002;34:592–5.](http://f1000.com/work/bibliography/6293962)
