# Supplementary material for: Socioeconomic status and stroke incidence, prevalence, mortality, and worldwide burden: an ecological analysis from the Global Burden of Disease Study 2017
Source: BMC Med. 2019 Oct 24;17:191. doi: 10.1186/s12916-019-1397-3 (PMC6813111; doi:10.1186/s12916-019-1397-3)
Supplement: Supplementary file 5 — Table S4. Socioeconomic Status and Stroke Outcome in Low- and Middle-Income Countries. (DOCX 132 kb) [file 12916_2019_1397_MOESM5_ESM.docx]

| Table S4. Socioeconomic status and stroke outcome in low- and middle-income countries. | | | | | | | | |
| --- | --- | --- | --- | --- | --- | --- | --- | --- |
| **Reference** | **Location** | **Study Type** | **Data Collection/Study Duration** | **Patient Group (N; age)** | **SES Determinants** | **Adjusted Risk Factors** | **Stroke Type** | **Results/ Remarks** |
| **Copstein et al. 2013** [**[26]**](http://f1000.com/work/citation?ids=2599139&pre=&suf=&sa=0) | Brazil | Population-based cross- sectional | July– December 2009 | 3,391; >20 years age | Marital status, education | Sex, age, hypertension, smoking | Stroke subtypes were not considered | The group with 9 to 11 years of education was statistically significant as a protective factor against stroke (PR 0.6, CI 0.4–0.9) and marital status widower was a significant risk factor of stroke (PR 1.4, CI 1.0–1.9) |
| **Engels et al. 2014** [**[27]**](http://f1000.com/work/citation?ids=2478890&pre=&suf=&sa=0) | Morocco | Population-based cross- sectional | November 2008– April 2009 | 60,031; >15 years age | Occupation, education | Sex, age, hypertension, diabetes; | Haemorrhagic stroke, ischaemic stroke, subarachnoid haemorrhage | The relationship between stroke and SES was nonlinear, with individuals from both the poorest (mainly rural) and richest (mainly urban) households having a lower prevalence of stroke as compared to individuals with medium wealth level |
| **Fitzpatrick et al. 2012** [**[28]**](http://f1000.com/work/citation?ids=2637095&pre=&suf=&sa=0) | Vietnam | Population-based cross- sectional | July 1– December 30, 2010 | 1,621; 35–93 years age (mean 52 years) | Urban/rural, education | Sex, age, diabetes, high cholesterol, hypertension, smoking, obesity, alcohol, physical activity | Stroke subtypes were not considered | Rural residence and not education were significantly associated with the presence of non-visual stroke symptoms |
| **Jin et al. 2012** [**[29]**](http://f1000.com/work/citation?ids=2599170&pre=&suf=&sa=0) | China | Hospital- based cohort | During 2006 | 6,102; <65 years and ≥65 years age | Income, marriage, living alone, occupation | Sex, age, diabetes, hyperlipidemia, alcohol, atrial fibrillation | Ischaemic stroke, haemorrhagic stroke, unknown type | Poor household and SES were unrelated to prehospital delay post-stroke |
| **Kamal et al. 2014** [**[30]**](http://f1000.com/work/citation?ids=2599341&pre=&suf=&sa=0) | Pakistan | Hospital- based case- control | 2007–2010 | 385; >18 years age (mean age of 62 years for cases controls) | Income, employment | Sex, age, hypertension, diabetes, dyslipidemia, tobacco use | Intracranial atherosclerotic disease- related stroke | Participants with no employment or who were retired were at an increased risk of stroke. Moreover, individuals with a monthly income of more than 15,000 Pakistani rupees had a higher risk of stroke than to those who earned less than 15,000 Pakistani rupees. |
| **Kumar et al. 2015** [**[31]**](http://f1000.com/work/citation?ids=2597293&pre=&suf=&sa=0) | India | Hospital- based case- control | February 2009– February 2012 | 448 (224 cases and 224 controls); mean age of 53 (SD 14) years for cases and controls | Household asset index for determining the socioeconomic status, education | Sex, age, hypertension, diabetes, dyslipidemia, smoking | Ischaemic stroke, large vessel stroke, small vessel stroke | The low economic status was independently associated with the risk of ischaemic stroke after adjustment for demographic and risk factor variables (OR 2.8; CI 1.2– 6.3) |
| **Menon et al. 2015** [**[32]**](http://f1000.com/work/citation?ids=1590201&pre=&suf=&sa=0) | India | Population-based cross- sectional | June 2010– February 2012 | 84,456; >18 years age (mean 44 years for both men and women) | Above/below poverty line | Sex, age, hypertension, diabetes, dyslipidemia, Smoking | Stroke subtypes were not considered | After adjustment for stroke risk factors, below the poverty line was associated with higher risk of stroke (OR 1.3, 1.0–1.7; p 0.02) |
| **Pandian et al. 2011** [**[33]**](http://f1000.com/work/citation?ids=2649478&pre=&suf=&sa=0) | India | Hospital- based cohort | March 2008– September 2009 | 448; 16–96 years age (mean 59 years) | Education, occupation, nutrition | Sex, age, smoking | Ischaemic stroke, haemorrhagic stroke | Premorbid undernutrition (OR 2.0), unemployment (OR 1.7) and lower education (OR 1.3) were independent predictors of poor outcome |
| **Tang et al. 2015** [**[34]**](http://f1000.com/work/citation?ids=2597297&pre=&suf=&sa=0) | China | Population-based cross- sectional | May 2009– August 2010 | 14,424 rural; >40 years age | Income, education | Sex, age, smoking, alcohol, hypertension, diabetes, obesity | Stroke subtypes were not considered | High-income villages were at higher risk of stroke (RR 1.7, CI 1.1–2.6) than lower-income villages |
| **Xu et al. 2008** [**[35]**](http://f1000.com/work/citation?ids=2478924&pre=&suf=&sa=0) | China | Population- based cross- sectional | October 2000– March 2001 | 29,340; >35 years age | Income | Sex, age, area of residence, obesity, cigarette smoking, alcohol consumption, diabetes, high blood pressure | Stroke subtypes were not considered | An elevated prevalence of stroke was associated with increasing levels of family average income |
| **Zhou et al. 2006** [**[36]**](http://f1000.com/work/citation?ids=2597299&pre=&suf=&sa=0) | China | Hospital- based cohort | August 1999– August 2002 | 806; mean age 71.0 (SD 11.2) years | Education, occupation, income, housing space | Sex, age, cigarette smoking, hypertension, diabetes mellitus, hypercholesterolemia, atrial fibrillation, myocardial infarction, prior transient ischaemic attack | Ischaemic stroke | Occupation, taxable income and housing space were significantly associated with three-year mortality after first-ever stroke |
| **Abbreviations**: CI, 95% confidence interval; N, number; OR, odds ratio; PR, prevalence ratio; RR, relative risk; SD, standard deviation; SES, socioeconomic status.  **Note**: We searched PubMed/Medline as of 1 March 2018 and the references of the retrieved articles for recent population-based or hospital-based cohorts (published in English) studying the association between SES and stroke. We used the MeSH terms “Socioeconomic Factors”, “Stroke”, “Adult”, “Cohort Studies”, “Risk Assessment”, “Incidence”, “Community Health Planning”, "Home Care Services, Hospital-Based", “Smoke”, “Alcohols”, “Obesity”, "Diabetes Mellitus", "Hyperlipidemias", "Air Pollution", "Atrial Fibrillation", "Stress, Psychological", "Atrial Fibrillation", “Malnutrition”, and "Nutrition Surveys", in addition to their entry terms and synonyms in LICs, LMICs, UMICs, and HICs (according to the 2017 World Bank classification of countries and regions [[3]](http://f1000.com/work/citation?ids=6309697&pre=&suf=&sa=0)). Inclusion criteria included: Type of study: cross-sectional case-control analysis of population-based or hospital-based nationwide longitudinal cohorts with no date or language limitations; Location of study: low- and middle-income countries based on the 2017 World Bank classification; Types of participants: patients of any age with a clinical diagnosis of stroke; Types of studied variable: SES with any socioeconomic measures; Types of adjusted variables: both sexes and age, along with one or more of the stroke modifiable risk factors; Types of outcome: stroke incidence or post-stroke-care/mortality within a predefined period in a longitudinal setting. Exclusion criteria included: Studies not fulfilling the inclusion criteria; Studies with unclear adjustment of stroke risk factors and covariates; Studies of cardiovascular disease with indefinite division of stroke related analysis. We retrieved 422 citations for title/abstract review; 394 citations were excluded based on selection criteria; 28 studies remained for the fulltext review, 11 of which were finally selected for data extraction. | | | | | | | | |
